# Translation dysregulation in neurodegenerative diseases: a focus on ALS

**DOI:** 10.1186/s13024-023-00642-3

**Published:** 2023-08-25

**Authors:** Shaopeng Wang, Shuying Sun

**Affiliations:** 1 Department of Physiology and Brain Science Institute, Johns Hopkins University School of Medicine, Baltimore, MD 21205 USA; 2 The Solomon H. Snyder Department of Neuroscience, Johns Hopkins University School of Medicine, Baltimore, MD 21205 USA; 3 Department of Pathology, Johns Hopkins University School of Medicine, Baltimore, MD 21205 USA

**Keywords:** ALS, Neurodegeneration, Translation regulation, RNA binding protein, Translation initiation, Translation elongation, Frameshifting, Ribosome quality control, Repeat expansion, RAN translation, Localized translation

## Abstract

RNA translation is tightly controlled in eukaryotic cells to regulate gene expression and maintain proteome homeostasis. RNA binding proteins, translation factors, and cell signaling pathways all modulate the translation process. Defective translation is involved in multiple neurological diseases including amyotrophic lateral sclerosis (ALS). ALS is a progressive neurodegenerative disorder and poses a major public health challenge worldwide. Over the past few years, tremendous advances have been made in the understanding of the genetics and pathogenesis of ALS. Dysfunction of RNA metabolisms, including RNA translation, has been closely associated with ALS. Here, we first introduce the general mechanisms of translational regulation under physiological and stress conditions and review well-known examples of translation defects in neurodegenerative diseases. We then focus on ALS-linked genes and discuss the recent progress on how translation is affected by various mutant genes and the repeat expansion-mediated non-canonical translation in ALS.

Amyotrophic lateral sclerosis (ALS) is an adult-onset progressive neurodegenerative disease mainly affecting motor neurons [1]. In the United States, around 5.2 people per 100,000 were diagnosed and the number was higher in whites, males, and people over 60 years old [2]. Until now there is no cure for it, and it usually leads to death within five years from onset. Sporadic ALS (sALS) accounts for 90% of all ALS cases and the other 10% is identified as familial ALS (fALS) with autosomal dominant inheritance [3]. Many cellular pathways have been suggested to contribute to the disease etiology, including mitochondrial damage, protein aggregation, excitotoxicity, nuclear pore defects, RNA dysregulation, etc.

RNA translation is tightly controlled in eukaryotic cells to regulate gene expression and maintain proteome homeostasis, which is important for cell function and survival [4]. RNA binding proteins (RBPs) play a crucial role in translation regulation through binding to mRNAs and recruiting corresponding regulating components [5, 6]. As mutations or pathology of multiple RBPs have been found to associate with ALS [7, 8], translational defect is a critical layer of RNA dys-metabolism underlying disease pathogenesis. Additionally, translation is also modulated by signaling pathways that sense various stimuli, including environmental and intracellular stresses, such as oxidative stress, ER stress of unfolded protein response, metabolism defects. These pathways are closely related to aging and neurodegeneration [9, 10]. It is likely that there is a complex interplay between the different mechanisms and RNA translation. Furthermore, besides canonical translation, an abnormal repeat-associated non-AUG (RAN) translation occurs in C9ORF72-linked ALS, which causes the production of toxic dipeptide repeat (DPR) proteins [11–14].

In this review, we will first introduce the general mechanisms of translational regulation, and examples of translation defects in neurodegenerative diseases. We will then focus on ALS-associated genes and discuss the recent progress in understanding the dysregulated translation in ALS.

## Translation

**Fig. 1:**
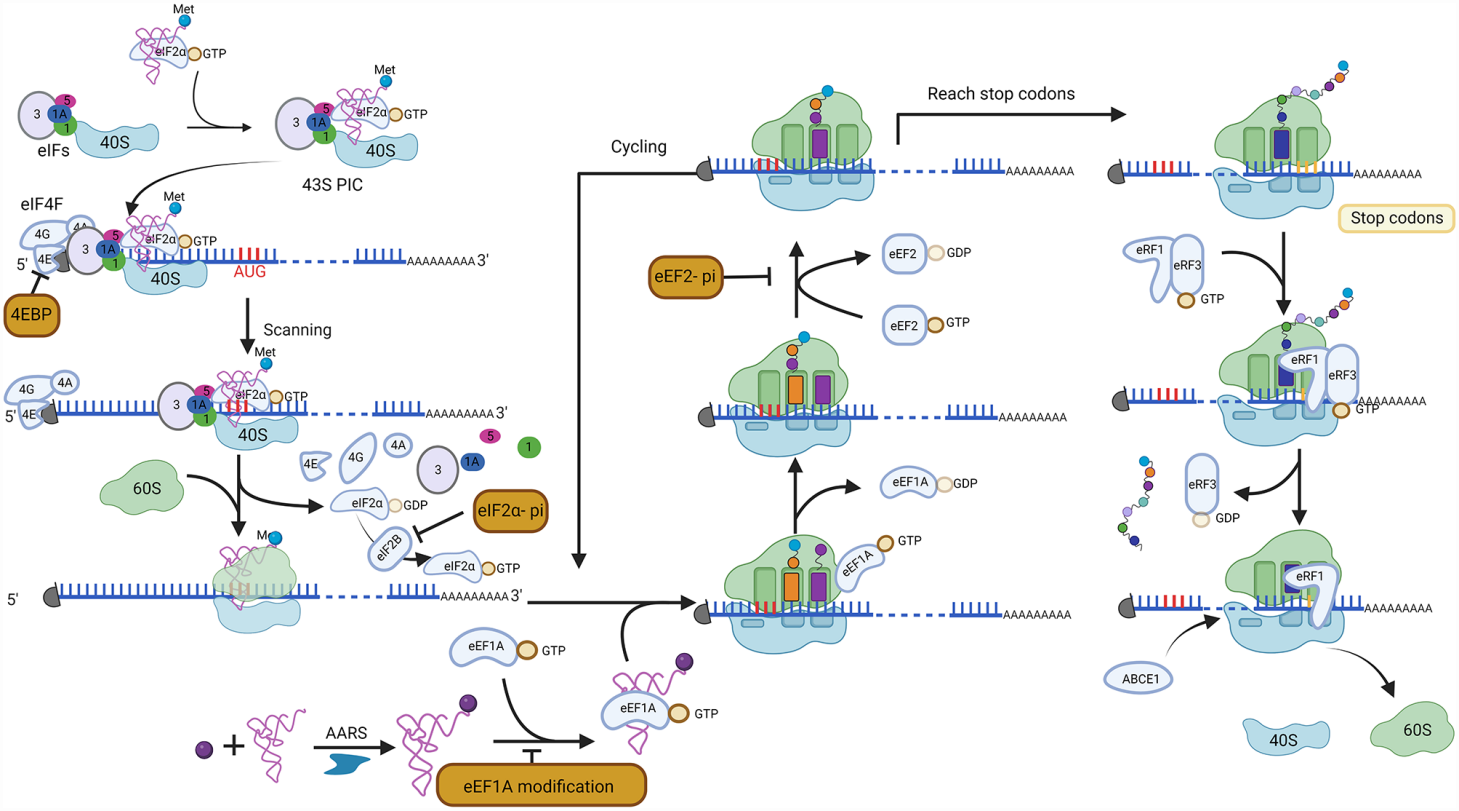
Overview of the canonical translation. During the initiation stage of translation, the 40S ribosomal subunit associates with various eIFs and the ternary complex to form the 43S PIC. The eIF4F complex recruits 43S PIC to the 5’ cap of mRNAs, forming the 48 S initiation complex. The recruitment process can be hindered by the eIF4E binding proteins (4EBPs), which disrupt the formation of the eIF4F complex. Once attached to mRNAs, the initiation complex scans the mRNA in the 5’ to 3’ direction to locate the start codon. Upon recognition of the start codon, initiation factors are released from the complex, and the 60 S large ribosomal subunit is recruited to assemble the 80 S ribosome. eIF2B catalyzes the reactivation of eIF2 by converting to its GTP-bound form, which can be inhibited by phosphorylated eIF2α. During translation elongation, eEF1A1 delivers cognate aminoacylated tRNA to the ribosome through base pairing between the codon and anticodon. With the assistance of eEF2, peptide elongation occurs as the ribosome translocates along the mRNA. Elongation continues until the ribosome reaches the stop codon. In eukaryotes, the termination of translation is mediated by the eRF1 and eRF3 complexes. These complexes play a role in the release of the nascent peptide, and subsequently, ABCE1 is recruited to the complex to facilitate the splitting of ribosomal subunits for recycling

Translation is the last step in the flow of genetic information which involves the decoding of the triplet codons in the mature mRNAs and the synthesis of corresponding proteins by ribosomes. It is one of the most complex and fundamental processes in cells and can be broadly divided into three steps: initiation, elongation, and termination [15] (Fig. 1). Initiation refers to the process that 80S ribosomes are procedurally assembled at the start codon (AUG) of mature mRNAs, promoted by multiple eukaryotic initiation factors (eIFs). The 40S ribosomal subunit first associates with eIF1, eIF1A, eIF3, and eIF5 and then assembles with the ternary complex which comprises eIF2, GTP, and the initiator tRNA (Met-tRNA_i_^Met^) to form the 43S pre-initiation complex (PIC). In canonical translation, the 43S PIC is then recruited to the 5’ end of mRNAs through the cap-binding complex eIF4F to form the 48 S initiation complex, which begins to scan the mRNA in the 5’ to 3’ direction until it reaches the start codon and establishes the codon-anticodon base pairing. The eIF2-bound GTP is then hydrolyzed, eIFs are released from the complex, and the 60 S large ribosomal subunit is recruited to assemble the 80 S ribosome at the start codon, which marks the end of the initiation phase [16, 17](Fig. 1).

After the assembly of 80 S ribosome, the initiator tRNA is in the P (peptidyl)-site of the ribosome. Elongation begins with the delivery of cognate aminoacylated tRNA to the A (aminoacyl)-site of the ribosome by a ternary complex formed with eukaryotic elongation factor 1 A (eEF1A), GTP and aminoacyl-tRNA (Fig. 1). Stimulated by the base pairing between the codon and anticodon, eEF1A-bound GTP is hydrolyzed and eEF1A-GDP is released. A peptide bond is formed between the new amino acid in the A site and the previous amino acid in the P site, transferring the nascent peptide from the P site to the A site. EEF2-GTP promotes the translocation of the tRNA from the P-site to E-site and the shifting of the next mRNA codon into the A-site. Following the release of the deacylated tRNA and the eEF2-GDP from the ribosome, the next cycle of elongation commences [18](Fig. 1). An important process in parallel with elongation is the synthesis of aminoacyl-tRNA. Aminoacyl-tRNA synthetases (AARSs) are the essential enzymes that catalyze the esterification of a tRNA to its cognate amino acid. AARSs are the only enzymes capable of implementing the genetic code, therefore critical in maintaining the translation fidelity [19](Fig. 1). Elongation continues until ribosome reaches the end of the coding sequence and a stop codon (UAA, UAG, or UGA) occurs in the A-site. At this point, translation goes into the final step called termination.

Termination is mediated by eukaryotic release factor 1 (eRF1) and eRF3. The ternary complex formed by tRNA-shaped eRF1 [20] and GTP-bound eRF3 recognizes the stop codon in the ribosome decoding center and binds to the A-site. After the hydrolysis of GTP, eRF1 is accommodated in the peptidyl-transferase center (PTC) and induces the release of the nascent peptide chain [21]. Next, ABCE1 is recruited to eRF1 and splits the 40 and 60 S ribosomal subunits for recycling (Fig. 1).

## Translational regulation and dysfunction in neurological diseases

### Translation initiation regulation

Regulating protein synthesis at the level of translation has obvious advantages over other layers of manipulation. As the final step of protein synthesis, translational regulation allows more immediate change on protein level from pre-existing mRNA. It enables cells to respond rapidly to stimuli. From the perspective of energy-consuming, translation regulation is much more efficient. As described above, every step of translation requires the usage of high-energy phosphate bonds. Indeed, it is estimated that around 30–50% of the cellular energy is consumed by translation [22–24]. Reducing protein levels by stopping the translation is, therefore, more energy-saving than inducing protein degradation. In cases where proteins need to be localized to function, it is also faster [25, 26] and more cost-effective in controlling localized mRNA translation than transporting proteins synthesized elsewhere [25–27].

Translational control happens at the levels of both global and specific mRNAs. The initiation phase is the rate-limiting step where most regulation is exerted. Global regulation mainly occurs through influencing the activity of general initiation factors. The cap-binding protein eIF4E is important for cap-dependent translation initiation. The post-translational modifications of eIF4E could influence translation, such as the phosphorylation of Ser209, which is generally believed to promote initiation [28]. The most well-studied regulation mechanism of eIF4E is through eIF4E binding proteins (4EBPs). 4EBPs bind to eIF4E and block the association between eIF4E and eIF4G, hence disrupting the formation of the eIF4F complex and inhibiting initiation (Fig. 1). Some 4EBPs, such as CYFIP1 (cytoplasmic FMR1-interacting protein 1), can recruit FMRP (fragile X mental retardation protein) to form a repressive complex to decrease initiation [29]. Activation of signaling pathways, such as mTORC1, leads to the phosphorylation of 4EBPs, causing their dissociation from eIF4E and thereby increasing translation [30].

eIF2 is a heterotrimer composed of three subunits: α, β, and γ. The α-subunit (eIF2α) is one of the most important targets for global translation regulation. The phosphorylation of eIF2α at the Ser51 residue increases its binding affinity to eIF2B [31], the guanine nucleotide exchange factor that catalyzes the reactivation of eIF2 by converting eIF2-GDP to eIF2-GTP [32]. Sequestration of eIF2B by phosphor-eIF2α prevents the recycling of eIF2 and thereafter attenuates global translation (Fig. 1).

### Integrated stress response

eIF2α phosphorylation is the core component of the integrated stress response (ISR), which is an evolutionarily conserved signaling network that helps cells maintain homeostasis under stress circumstances. There are four different kinases that catalyze the phosphorylation of eIF2α to reduce global translation. PKR (double-strand RNA-dependent protein kinase) is initially found to be activated by virus infection [33], but has also been found to be activated by ER stress, oxidative stress [34], high glucose [35] and metabolic stress [36]. PERK (PKR-like ER kinase) is an ER transmembrane protein, mainly activated by the accumulation of misfolded or unfolded protein in the ER lumen [37]. GCN2 (general amino acid control nonderepressible 2) is activated by amino acid deprivation via binding to the deacylated His-tRNA, as well as by UV irradiation [38], and glucose deprivation [39]. HRI (heme-regulated inhibitor) can be activated by many other triggers such as oxidative stress, osmotic stress, heat shock, and proteasome inhibition [40].

ISR plays important roles in aging and neurodegenerative diseases. For example, oxidative stress, ER stress, proteotoxic stress, and neuroinflammation are characterized in many neurodegenerative disorders including Alzheimer’s disease (AD), Parkinson’s disease (PD), ALS, frontal temporal dementia (FTD), and Huntington’s disease (HD) [41–43]. Indeed, elevated and/or dysregulated eIF2α phosphorylation has been extensively associated with those diseases [41]. The attenuation of global translation could lead to synaptic dysfunction and neuronal loss [44]. Consistent with this concept, it has been shown that inhibiting ISR could be neuroprotective [45]. For example, ISRIB (integrated stress response inhibitor), a compound that reverses the effect of eIF2α phosphorylation [46], rescues cognitive defects in AD mice [47]. Temporary treatment of old mice with ISRIB reverses age-related ISR activation and inflammatory profiles, rescues neuronal function, reverses spatial memory deficits and improves working memory [48]. Blocking ISR genetically or pharmacologically also has been shown to ameliorate cell death in animal or cell models of PD [49], prion disease [44], and ALS [50]. Counterintuitively, some studies also revealed the beneficial effects of ISR activation, including ALS [51–53], PD [54], and HD [45, 55]. It is likely the timing and dynamic balance of the response are critical for the phenotypes. The exact underlying effects of ISR in different neurodegenerative diseases and corresponding intervention strategies remain to be elucidated.

Mutations in genes encoding the five subunits of eIF2B cause the autosomal recessive inherited neurological disorder leukoencephalopathy with vanishing white matter (VWM) [56]. Loss of eIF2B function may impair protein synthesis (such as myelin) [57] and increase the vulnerability of cells to deal with stress [58]. It has been demonstrated that ISR was activated in glial cells of VWM patient cerebral white matter [59]. Under stress conditions, cells derived from VWM patients showed more severe translation inhibition and defects in translation recovery compared to cells derived from controls [58].

### Translation elongation regulation

Although initiation phase is the primary focus of translation regulation, emerging evidence demonstrates that elongation also plays important roles. Modifications of elongation factors such as eEF1A1 and eEF2 influence translation elongation broadly (Fig. 1). Residue Ser300 of eEF1A1 is important for its binding to aminoacyl-tRNA. Phosphorylation of Ser300 decreases the binding affinity and inhibits translation [60]. Phosphorylation of eEF1A1 at other sites such as the residue Ser396 also has been shown to inhibit translation [61]. Lysine methylation of eEF1A1 regulates translation by influencing the ability of eEF1A to interact with various aminoacyl-tRNAs or its interaction with the translating ribosome [62]. Phosphorylation of eEF2 decreases its binding affinity with ribosomes, therefore inhibiting translation [63, 64] (Fig. 1).

Complete loss of eEF1A2, an isoform of eEF1A which is selectively expressed in neurons and muscles [65], leads to neurodegeneration and muscle wasting in mice [66]. In vertebrates, the expression of eEF1A1 decreases in neurons and muscles during postnatal development and its function is taken over by increased expression of eEF1A2. However, both eEF1A isoforms are lost due to the spontaneous autosomal recessive mutation of eEF1A2 in wasted mice, which leads to motor neuron degeneration and muscle wasting [67]. Additionally, eEF1A2 mutations have also been shown to influence translation fidelity, such as increasing amino acids misincorporation and promoting frameshift [68]. De novo missense mutations in eEF1A2 have also been identified in patients with diverse neurodevelopmental syndromes such as intellectual disability [69], epilepsy [70], autistic behavior [71], and Rett syndrome-like (RTT‐like) phenotype [72], etc. Taken together, dysregulated translation elongation caused by eEF1A2 mutations is a key contributor to neurodegeneration.

Mutations in aminoacyl-tRNA synthetases (AARSs), the essential enzymes that charge tRNAs with cognate amino acids, cause many diseases including neurological disorders. Charcot-Marie-Tooth disease (CMT), one of a group of disorders that cause damage to the peripheral nerves, is the first disease that linked to mutations in AARSs. Until now mutations in at least six AARSs (GARS, YARS, AARS, MARS, HARS, and WARS) have been linked to CMT [73]. Multiple lines of evidence including the mono-allelic nature of the CMT-causing mutations indicate gain-of-toxic-function disease mechanisms. Take GARS as an example, overexpression of CMT-mutant GARS recapitulates several hallmarks of human disease [74, 75]. The mutant GARS impairs global protein translation in motor and sensory neurons independent of its aminoacylation activity [74]. It was recently shown that CMT-mutant GARS influences translation elongation by sequestering cellular tRNA^Gly^, which depletes available tRNA^Gly^ and results in ribosome stalling and translation reduction [76, 77]. Prolonged ribosome stalling may activate ISR through the GCN2 pathway [78]. Indeed, the ISR related genes were found to be upregulated in pre-disease onset mice [79]. Genetic deletion or pharmacological inhibition of GCN2 alleviates ISR and neuropathy in the CMT-mutant GARS mice [79]. Restoring translation by overexpressing wild type tRNA^Gly^ in fly and mice models prevents ISR activation and rescues peripheral neuropathy [76]. Thus, the translation defects in CMT due to the sequestration of tRNA by mutant AARS can further activate ISR, which in turn contributes to pathophysiology [76, 79, 80].

Mice with homozygous N-ethyl-N-nitrosourea (ENU)-induced mutation *nmf205* develop cerebellar, hippocampal, cortical, and retinal neuron degeneration. This was identified to be caused by a null mutation in the gene encoding GTPBP2, a translational GTPase [81]. Interestingly, the severity of the neurodegeneration phenotype varies tremendously depending on the genetic background. Mutation in an arginine tRNA gene, *n-Tr20*, exacerbates the neuronal death when combined with *Gtpbp2* mutation [81]. This tRNA gene is specifically expressed in the nervous system. Loss of function results in increased ribosome pausing during translation elongation. The stalled ribosomes could not be released without GTPBP2, which further activates ISR through GCN2 and accelerates neurodegeneration [78]. Overall, these indicate that mutations in tRNA and elongation factors may result in global translation defects and stress signaling activation. And due to tissue specific expression, certain mutations can influence neuronal cells specifically and cause neurodegeneration.

### Localized translation in neurons

Asymmetric mRNA localization and localized translation provide the opportunity to fine-tune localized protein concentration which plays an important role in development [82], cell fate determination [83], cell migration [84], etc. Localized translation is of particular importance in neurons due to their unique morphology and complex networks. It is believed to have fundamental roles in many neuronal processes including neuronal development, axonal maintenance, synapse function and synaptic plasticity [85, 86]. Thousands of mRNAs are identified in dendrites and axons of different types of neurons [87, 88] and found to be translated locally [89]. mRNAs are selectively delivered to different cellular compartments through the coordination of cis-acting elements and trans-acting RBPs [26]. It has been demonstrated that a single mRNA can recruit multiple RBPs which may further recruit other regulatory proteins and assemble into transport mRNP granule. Once assembled, mRNPs are transported to distal compartments through direct binding to motor proteins to transport on cytoskeletons [90, 91] or through hitchhiking on other moving organelles such as endosome, mitochondria [86, 92] and lysosome [93]. Proteins in mRNP granules also protect mRNA from degradation and participate in the regulation of mRNA translation. The translation of mRNA is believed to be usually repressed during transport and may be activated upon the arrival at its destination and local stimulation.

Many neurological disease-related proteins have been shown to participate in the process of mRNA transport and localized translation in neurons. The survival motor neuron (SMN) protein whose loss is linked to the neuromuscular disease spinal muscular atrophy (SMA) is recently found to participate in mRNA transport [94]. It has been demonstrated that SMN facilitates the binding of ZBP1 to *ACTB* mRNA which is important for the proper transport of *ACTB* mRNA [95]. SMN depletion significantly reduces axonal mRNAs [96] including those associated with axon growth and synaptic activity [97]. KIF5A, an ALS-associated gene [98], is a member of the Kinesin superfamily proteins (KIFs) which mediate the transport of cargos along microtubules. It was also shown to be involved in the delivery of RNA in neurons [99]. Many neurological diseases related proteins including hnRNPA, hnRNPU, FMRP, FUS, and staufen are identified in KIF5A-related mRNA transport granule indicating their important role in neuronal RNA transport [100].

### Unconventional translation of expanded RNA repeats

Expansions of short nucleotide sequence repeats account for more than 50 neurological or neuromuscular diseases [101]. The pathogenic mechanism among those diseases varies, influenced by repeat sequence, length, location, and the genetic context. One special phenomenon of the repeat expansion is the non-canonical translation of the repeat-containing RNA, recognized as repeat-associated non-AUG (RAN) translation. The translation of the RNA repeats in all possible reading frames generates various poly-peptide proteins, which contribute to disease pathogenesis [102]. RAN translation is first described in CAG·CUG expansion-associated spinocerebellar ataxia type 8 (SCA8) and myotonic dystrophy type 1 (DM1) in 2011 [103]. Since then, RAN translation has been investigated and detected in many microsatellite expansion-associated diseases [104] including fragile X-associated tremor/ataxia syndrome (FXTAS) (CGG•CCG) [105], myotonic dystrophy type 2 (CCTG•CAGG) [106], spinocerebellar ataxia type 31 (SCA31) (TGGAA•TTCCA) [107], SCA36 (TGGGCC•GGCCCA) [108], Huntington’s disease (CAG•CTG) [109], and C9ORF72-ALS/FTD (GGGGCC•GGCCCC) [110–112]. RAN translation is found to occur in a surprising variety of RNA contexts, including untranslated regions (UTRs), protein-coding open reading frames (ORFs), and introns. The secondary structures of the expanded RNA repeats are important for the non-canonical translation initiation that does not require the AUG start codon, and sometimes the 5’-cap as well. Increasing studies revealed molecular mechanisms and genetic modifiers that can regulate the translation efficiency of the repeat expansion. It is noted that there has been debate about whether RAN translation is truly a novel translation mechanism, or such non-canonical translation also occurs at low levels in regular RNA sequences. It is likely the repeat expansion increases the chance of an existing non-canonical event. Additionally, the sequences upstream of the repeats can influence the initiation mechanisms. Some of the reading frames use near-cognate start codons or in-frame AUG to initiate translation, thus not all the poly-peptides are produced by RAN translation. Nevertheless, it is important to determine the factors or pathways that can regulate the translation of repeat RNA, as this will provide strategies to reduce the toxic protein products generated from the repeat expansion. We will focus on the C9ORF72 hexanucleotide repeat expansion, which is the most common genetic cause of ALS and FTD, in this review.

## Translation defects in ALS

### C9ORF72

#### Translation of the expanded repeats

Hexanucleotide GGGGCC repeat expansion in the first intron of the *C9ORF72* gene is the most common genetic cause of both ALS and FTD [113, 114]. Through bi-directional transcription, both sense (GGGGCC) and antisense (CCCCGG) repeats-containing RNA are synthesized and used as the templates for translation to produce five different DPR proteins (poly-GA and poly-GR from sense repeats, poly-PA and poly-PR from antisense repeats, and poly-GP from both strands) [12–14, 115]. DPR pathology is a hallmark of C9ORF72-ALS/FTD and the toxicity of DPRs has been extensively studied in both cell culture and animal models [116]. An approach to decrease the levels of these toxic dipeptides by inhibiting their production holds potential therapeutic promise.

Many advances have been made on *C9ORF72* repeat RNA translation mechanisms and regulatory pathways. The traditional method is using ensembled assays, such as luciferase, fluorescence proteins or short tags fused to the repeats to monitor the DPR levels translated from the repeats. This allows straightforward test and identification of novel modifiers or pathways that can modulate the DPR production. But one limitation is that the final protein product level can be influenced by many RNA/protein regulation steps besides translation, thus it is sometimes challenging to elucidate the exact molecular mechanisms. Alternatively, recently developed imaging approaches allow direct visualization of single RNA molecule dynamics in live cells, which can be used to assess translation directly. Finally, the measurement of endogenous DPRs in patient cells is critical to validate the findings from reporter systems.

#### The GGGGCC repeat-containing RNA template for RAN translation

Earlier examples of RAN translation occur on repeats in the UTR or ORF regions of mature mRNAs, which are naturally exported to cytoplasm for translation. However, the *C9ORF72* expanded repeats are located in the intron. Both the spliced intron and not fully processed intron-containing pre-mRNA are normally excluded from cytoplasm under multiple surveillance mechanisms [117, 118]. Therefore, the production of DPRs from the C9ORF72 GGGGCC repeats also depends on the unusual nuclear export of the intronic repeat RNA besides the non-canonical translation.

The single-molecule Fluorescence in Situ Hybridization (smFISH) provides an opportunity to directly visualize the spatial localization of single RNA transcripts in cells. The intron- and exon-containing molecules can be visualized simultaneously using orthogonal RNA tags (MS2 and PP7), which allows the examination of the molecular identity and spatiotemporal dynamics of the repeat RNA [119]. It is demonstrated that the GGGGCC repeat-containing RNA transcripts in the cytoplasm are spliced introns, but not unspliced pre-mRNAs. This was further confirmed in patient-derived cells by smFISH using probes targeting endogenous *C9ORF72* intron 1 or all exons. Furthermore, by combining smFISH with exonuclease RNase R treatment, it was suggested that the cytoplasmic repeats containing introns mainly existed in a circular form, due to the defective debranching of spliced lariat intron induced by the GGGGCC repeats, which is believed to be more stable compared with linear RNAs [119]. It is noted that the intron retention isoform has been reported to be elevated by the repeats [119–121]. Although translation on pre-mRNA was not observed using the reporters [119], there is likelihood that low abundance of transcripts with retained intron are localized to cytoplasm and subjected to translation from the endogenous repeats.

#### Modifiers of nuclear export of GGGGCC repeat-containing RNA

Various approaches have been used to identify modifiers of the repeat RNA metabolisms. Many RBPs have been reported to modulate the repeat RNA nuclear export and translation. Through RNA-affinity pulldown assay, several RBPs with functions in RNA transport such as serine and arginine-rich splicing factors (SRSF), ALYREF, and transcription-export complex (TREX) subunits were identified as GGGGCC repeat RNA binding proteins [122, 123]. The nuclear RNA export factor 1 (NXF1) and its cofactor NXT1 are the key components of TREX and TREX-2 complexes, which predominantly mediate mRNA export [124]. In an unbiased CRISPR-Cas9 knockout screen to identify genetic modifiers of DPR production, several genes in TREX and TREX-2 were identified as enhancers of DPR production [125]. Knockdown components of this pathway can reduce the DPR levels [125]. The single-molecule imaging approach also provided direct evidence that reduction of the NXF1 pathway preferentially inhibits the export of GGGGCC-containing spliced circular intron while the linear mRNAs are only affected modestly [119]. It is likely that specific RBPs bind on the repeats and mediate the interaction with the TREX complexes. Indeed, SRSF1 has been shown to act as an adaptor that directly binds to GGGGCC repeat RNA and interacts with NXF1 to trigger the export [126]. Altering the phosphorylation of SRSF1 influences the nuclear export of GGGGCC repeat RNA [127]. There are possibly other export receptors and adaptors that participate in the export of GGGGCC repeat-containing intron. As reduction of repeat RNA export can decrease the DPR accumulation, it is of great interest to identify additional export receptors and adaptors, especially those that have higher specificity on the repeats, and assess their therapeutic values.

#### Translation initiation mechanisms of intronic GGGGCC repeats

Although the canonical translation initiation requires 5’ cap, many viral RNAs and some cellular RNAs can start translation in a cap-independent manner, using the internal ribosome entry site (IRES), which usually contains complex RNA structures that directly recruit certain translation initiation factors to start translation [128]. It has been shown that the translation of CGG repeats located in the 5’ UTR of *FMR1* requires the 5’ 7-methylguanosine (m^7^G) cap on the mRNA [129]. However, as the spliced circular intron is exported to the cytoplasm [119], the cap-independent translation initiation is important for C9ORF72 repeat expansion. The active translation of spliced intron has been directly observed in live-cell using the single-molecule imaging of nascent peptides (SINAPS) technology in which the translation dynamics is monitored by SunTag epitopes [119]. Studies using ensemble approaches also suggest that the translation of GGGGCC repeats can initiate from RNA transcript without the 5’ cap, although the efficiency is lower than that on the capped repeat RNA [130–134].

**Fig. 2:**
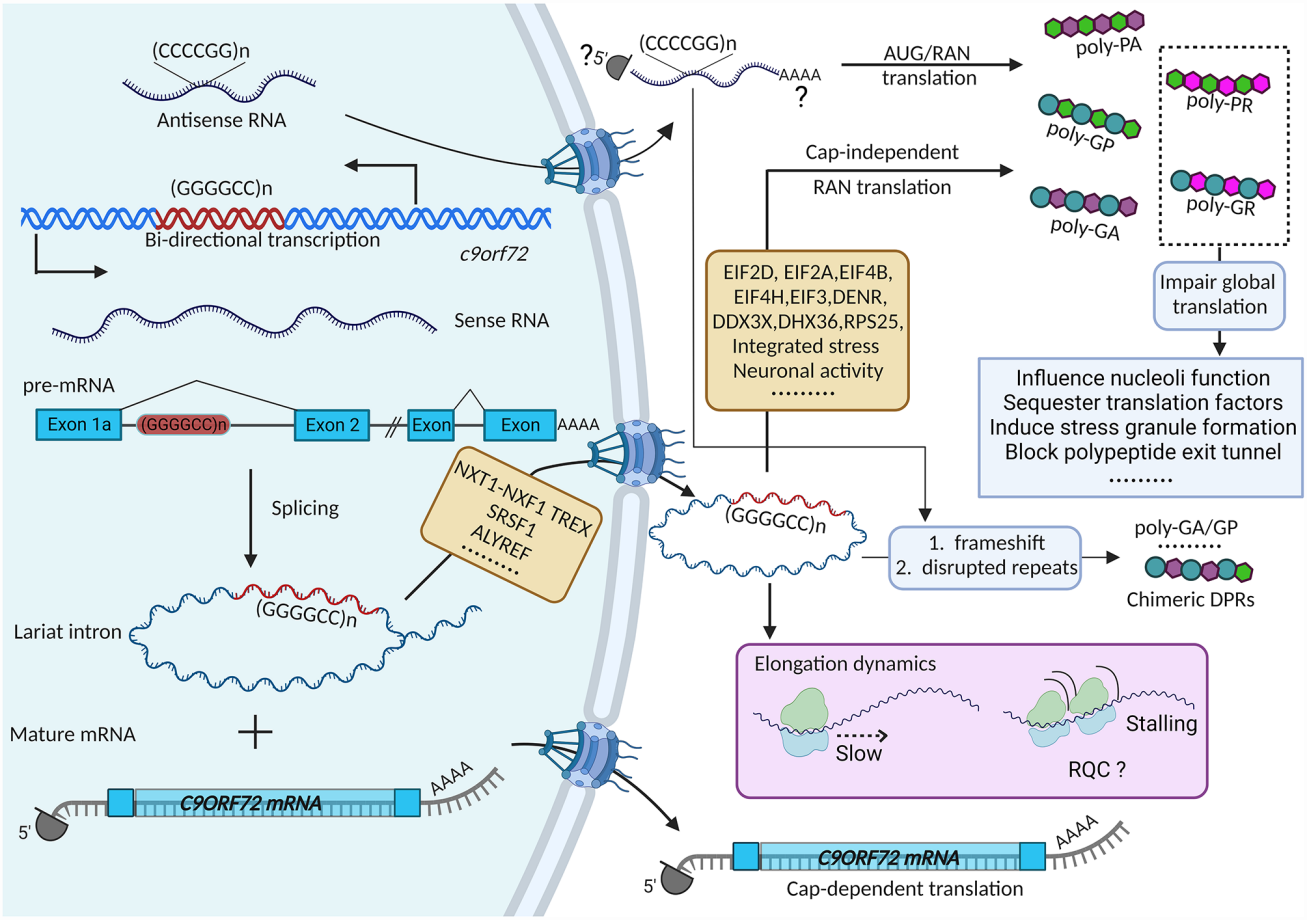
Overview of *C9ORF72* repeat RNA translation. First, through bi-directional transcription, both sense (GGGGCC) and antisense (CCCCGG) repeat-containing RNA transcripts are produced from the first intron of the *c9orf72* gene. The pre-mRNA containing the sense repeats will be processed in the nucleus to produce mature *C9ORF72* mRNA and GGGGCC repeat-containing lariat intron. The debranching of the lariat can be inhibited by the GGGGCC repeats. The repeat-containing intron is stabilized and exported to the cytoplasm in the circular form (which does not have Cap structure and poly-A tail). The NXT1-NXF1 pathway, as well as specific RNA-binding proteins (RBPs), play important roles in mediating the export of GGGGCC repeat-containing introns. The GGGGCC repeat-containing circular introns undergo repeat-associated Non-AUG (RAN) translation in all three reading frames to produce poly-GA, poly-GP, and poly-GR dipeptide repeats (DPR) proteins. Many RBPs, translation factors and signal pathways can regulate the translation initiation efficiency. The antisense transcripts likely contain 5’ Cap and 3’ poly-A, similar as regular mRNAs. Translation from all the three reading frames produce poly-GP, poly-PA, and poly-PR. Chimeric DPR proteins may be synthesized due to translational frameshift or disrupted repeat sequences. Due to the repeat sequences and RNA structures, reduced elongation speed and ribosome stalling may occur during the translation through the repeats, which could potentially activate the RQC pathway. Finally, the arginine-containing DPRs, including poly-GR and poly-PR, can impair global translation through different mechanisms

The small ribosomal protein subunit 25 (RPS25), a non-essential protein component of the small (40 S) ribosomal subunit which has been shown to facilitate the recruitment of 40 S ribosomal subunit to IRES RNAs [135, 136], can selectively regulate GGGGCC associated RAN translation. Knockdown or knockout of RPS25 significantly decreased the DPR level without affecting the repeat RNA abundance and global translation [137]. Eukaryotic translation initiation factor 3 F (eIF3F), a noncore subunit of the eIF3 complex, has been shown to bind to IRES and regulate the translation of hepatitis C viral (HCV) RNA. Knockdown of eIF3F caused a 43% reduction of poly-GP protein level [138]. Taken together, the evidence supports that the cap-independent translation initiation is an important contributor to RAN translation of GGGGCC repeats in C9ORF72-ALS/FTD (Fig. 2). It needs further exploration whether the intron retention transcripts, if exported to cytoplasm, undergo cap-dependent translation and how each contributes to DPR production. Nevertheless, the location and gene context of the repeat expansion are critical for understanding the repeat RNA processing pathway.

#### Modifiers of GGGGCC repeat translation initiation

Many translation factors have been shown to regulate GGGGCC associated RAN translation. In the *C. elegans* model of C9ORF72-associated ALS/FTD, functional loss of non-canonical initiation factor eIF2D but not eIF2A reduces DPR production and ameliorates lifespan and locomotion defects [139]. Depletion of eIF2A has been shown to decrease cap-independent RAN translation of GA [132] but has little effect on cap-dependent GA RAN translation [140] in HEK293 cells. Knockdown of DENR, another non-canonical initiation factor, inhibits GGGGCC-associated RNA translation and improves the survival of drosophila expressing expanded GGGGCC repeats [140]. Two other initiation factors, eIF4B and eIF4H, also have been shown to facilitate RAN translation of GGGGCC repeats to produce DPR proteins. Depletion of eIF4B and eIF4H rescues GGGGCC associated toxicity in fly [141].

As RNA secondary structure is important for RAN translation, RNA helicases are also implicated in RAN translation. The GGGGCC repeat RNA has been shown to form both hairpin and G-quadruplex structures [142, 143]. From a CRISPR-Cas9 screen, DDX3X was identified to reduce the RAN translation of GGGGCC repeats. The study suggests that DDX3X binds to the hairpin structure of the GGGGCC repeats and its helicase activity is essential for the translation repression. This indicates DDX3X alters the GGGGCC repeat RNA structure, which is important for the non-canonical translation initiation. Indeed, elevating DDX3X expression decreased DPR levels and improved the survival of C9ORF72-ALS patient iPSNs [125]. On the contrary, another helicase, DHX36 was reported to facilitate translation elongation through the GGGGCC repeat RNA, by binding to and unwinding the G-quadruplex formed by the GGGGCC repeats. Depletion of DHX36 in patient iPSNs decreased the levels of endogenous DPR proteins [144], and overexpression of DHX36 enhances RAN translation of GGGGCC repeat expansion [145]. Overall, it is likely that different helicases could bind to different secondary structures or affect different steps of repeat translation. The DPR production will be influenced by the combinatorial effects of multiple RBPs and translation factors (Fig. 2).

#### RAN translation regulation by cell signaling

The initiation of RAN translation can also be regulated by cell signaling pathways. As noted in the previous section, ISR is associated with many neurodegenerative diseases. The ISR activation leads to global translation repression, but a subset of mRNAs has increased translation instead, which usually use the non-canonical initiation mechanisms [146]. Using single-molecule live-cell imaging, sodium arsenite (oxidative stress inducer) treatment elevated RAN translation from the repeat-containing spliced intron as early as 7 min after drug application [119]. Indeed, the DPR levels were drastically upregulated upon various stress stimuli in multiple reporters [130, 131, 147]. Given that DPR proteins, such as GR, increase oxidative stress and DNA damage in cells, a positive feedback loop between RAN translation and ISR is established which could contribute to the irreversible cell death and neurodegeneration. Inhibition of eIF2α signaling pathway by two small molecule inhibitors, ISRIB and PERKi [148] (GSK2606414, PERK inhibitor), reduced the stress-induced upregulation of RAN translation [130]. PERKi has been shown to decrease poly-GA levels and rescue cell vulnerability in human C9ORF72-ALS/FTD brain organoid slice cultures [149]. Blocking PKR activation (presumably induced by repeat RNA structure) by metformin has also been reported to reduce RAN translation in the C9ORF72 BAC transgenic mice [150]. Overall, these studies indicate that the eIF2α signaling pathway is a promising therapeutic target to reduce the DPR production and toxicity.

In addition to ISR, changes in neuronal activity can also influence C9ORF72 GGGGCC RAN translation. Activation of glutamate receptors by various agonists increases RAN translation in both primary cortical neurons and in patient-derived iPSNs [147]. Elevating neuronal activity optogenetically also promotes DPR production [147]. Given the fact that age-dependent hyperexcitability and increased sensitivity to glutamate-induced excitotoxicity are common phenomena in C9ORF72-ALS/FTD patients [151–153], inhibiting neuronal activity could potentially reduce the toxic DPR production and provide protective efficacy.

#### Translation frameshift through the repeats

Ribosomal frameshifting is an evolutionarily conserved biological process that has been found in many organisms such as viruses, bacteria, and eukaryotes. Programmed ribosome frameshifting is used to produce distinct proteins from the same RNA, which is a fundamental mechanism for viruses to maintain their infection efficiency [154]. Frameshifting also happens in eukaryotic cells although it most often triggers nonsense mediated decay (NMD) to degrade the target mRNA due to the occurrence of premature stop codons after frameshift [155]. The secondary structure of mRNA may cause the pause of ribosomes during the translation elongation, and therefore is recognized as a modulator of frameshift [154]. Indeed, it has been demonstrated that both CAG•CTG and CGG•CCG repeats have a propensity to undergo frameshifting, resulting in the production of frameshifted proteins [156–158].

Chimeric GA:GP protein has also been detected in C9ORF72-ALS/FTD patient postmortem tissues including the frontal cortex and cerebellar cortex by an immunoassay using GA antibody for capture and GP antibody for detection [108]. This could be caused by frameshift during the translation of the GGGGCC repeats, or genetic interruptions in the repeat expansion [11, 108, 159] (Fig. 2). An upstream near-cognate CUG start codon has been identified as the translation start site of the GA reading frame [131, 134]. The in vitro translation assay showed that mutating CUG was sufficient to reduce the DPR levels of all the three reading frames, implicating that poly-GP and poly-GR are predominantly produced from frameshifting from the GA frame [134]. However, homozygous deletion of the intron region before the repeats, containing the CUG codon, ablates the production of poly-GA but not poly-GP and poly-GR in C9ORF72 iPSNs [160]. This supports that poly-GP and poly-GR are majorly generated independent of the GA frame instead of from frameshifting, in contrary to the previous study. The inconsistent results obtained using different model systems warrants further exploration.

As the pure repeated DPRs and the chimeric DPRs probably have different properties and toxicities, it is important to understand the efficiency of translation frameshifting on the repeats and to what extent it contributes to the generation of various DPR species. Recently a new technology that uses multicolor probes to visualize the translation of different reading frames of a single RNA provides the opportunity to directly quantify the frameshifting activities in live cells. The study using the established HIV-1 frameshift sequence revealed that only a small subset (8%) of translating RNAs showed robust frameshifting event [161]. Application of such technology will help determine the frameshifting frequency during the repeat RNA translation. Additionally, how frequent the genetic interruptions are present in the repeat expansion that can directly serve as the template for chimeric DPRs in patients is also a critical aspect. The long-read sequencing technique through the GGGGCC expansion will provide the answer. Finally, another challenging question is what is the proportion of the individual dipeptide in the chimeric products. It has been shown that a low amount of incorporation of a secondary poly-dipeptide did not have significant impact on the features of the primary poly-dipeptide [108]. What combination of the chimeric proteins can lead to different toxicity features and what species exist in patients need to be carefully explored when understanding the pathophysiological significance.

#### Translation elongation of different reading frames on the repeats

Once initiated, the translation of the repeats follows the canonical elongation mechanisms. The GGGGCC repeat-containing RNAs form sophisticated secondary structure [142, 143] and encode repetitive amino acids. This likely affects the translation elongation dynamics and causes ribosome stalling compared to non-repetitive sequences [133]. The different codon usage can also influence translation elongation [162]. Indeed, it has been shown that the arginine-rich DPRs encoded by randomized codons are stalled on ribosomes during translation [163]. Ribosome stalling is recognized as a trigger of ribosome-associated quality control (RQC) pathway [164]. It is speculated that translation of GGGGCC repeats, especially in the GR frame, can activate the RQC pathway. Consistent with this hypothesis, ZNF598, a protein that plays a critical role in RQC, has been identified as a modifier of poly-GR protein [165]. It is proposed ZNF598 promotes poly-GR degradation, although the detailed mechanism needs further investigation. In addition, other RQC factors, including Ltn1, VCP1, Pelota, and ABCE1, has also been shown to modulate poly-GR protein level in fly [166]. Two recent studies also indicate that ribosome stalling and RQC are closely related to the translation of poly-GR [167, 168]. Overall, these data implicate that the elongation of poly-GR may encounter ribosome stalling and be subjected to RQC regulation (Fig. 2). More direct evidence on translation dynamics will be needed in future work.

#### poly-GR/PR influence global translation

**Fig. 3:**
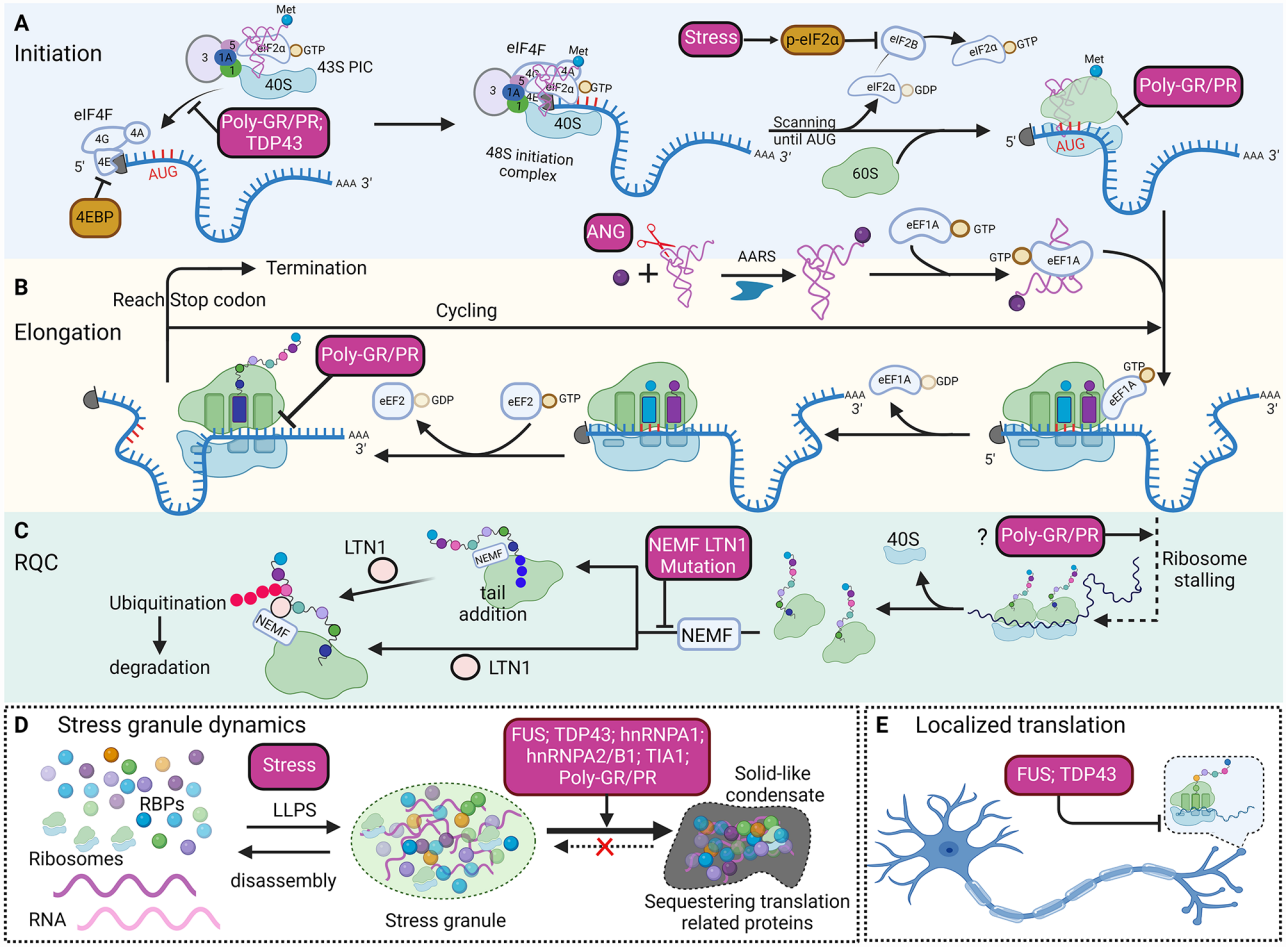
Overview of how translation regulation is affected by ALS-related genes. **(A)** The impact of ALS-related genes on translation initiation. Integrated stresses, which are frequently observed in ALS, induce eIF2α phosphorylation, leading to the sequestration of eIF2B and impairment of translation initiation. Poly-GR/PR and TDP-43 may result in the sequestration of translation factors, thereby affecting translation initiation. Poly-GR/PR can also bind to the polypeptide exit tunnel of the ribosome thus blocking translation initiation. **(B)** The influence of ALS-related genes on translation elongation. poly-GR/PR can bind to the polypeptide exit tunnel of the ribosome and inhibit translation elongation. Another important process for translation elongation is the charge of tRNAs with cognate amino acids by aminoacyl-tRNA synthetases. Under stress conditions, ANG may translocate to cytoplasm and cleave tRNAs which further inhibits translation. **(C)** When ribosome stalling occurs, ribosome quality control (RQC) pathway is activated to split the stalled ribosome subunits. NEMF participates in the recognition of obstructed large ribosomal subunit and recruits LTN1 E3 ligase to catalyze the poly-ubiquitination of the nascent peptide chain for degradation. NEMF also can add C-terminal tails to the nascent chains to facilitate its ubiquitination. NEMF and LTN1 mutations disrupt RQC pathway and cause ALS-like phenotypes in mice. As poly-GR/PR can bind to the polypeptide exit tunnel of the ribosome, they may cause ribosome stalling and trigger RQC pathway. **(D)** Under stress conditions, RBPs and mRNAs undergo LLPS (liquid-liquid phase separation) to form membrane-less condensates which are termed stress granules. The assembly and disassembly of stress granules are dynamic in response to stress stimuli. However, ALS-related mutations in many genes and poly-GR/PR influence the dynamics of stress granule formation and promote the transition of stress granules to solid-like condensates which may influence global translation by sequestering translation machinery. **(E)** ALS-related mutations in FUS and TDP-43 have been shown to impair localized translation in neurites

Multiple lines of evidence indicate that arginine-rich DPRs (poly-GR and poly-PR) can cause translation defects (Fig. 3). Earlier studies using short poly-dipeptides showed that poly-GR and poly-PR localize in nucleoli, where the ribosomes are produced and assembled [169, 170]. Two nucleolar proteins, NPM1 and NCL1, interact with poly-GR/PR in live cells. This impairs the dynamics and function of nucleoli [171]. Consequently, total rRNA is significantly reduced in poly-GR/PR expressing cells, which could presumably impair translation [171]. Several interactome studies in multiple in vitro and in vivo model systems further identified ribosomal proteins as the interactors of poly-GR and poly-PR [163, 171–174], even when poly-GR is predominantly localized in cytoplasm [163, 173]. Cytosolic poly-GR colocalizes with the translation initiation factors and ribosome proteins in AAV-GR_100_ mice and C9ORF72-ALS/FTD patient postmortem brain tissues [175], suggesting poly-GR might also affect translation with alternative mechanisms in addition to the effect on ribosome assembly in nucleoli. Several studies have shown that expression of poly-GR and poly-PR leads to reduced rate of protein synthesis [171, 172, 174, 175]. In vitro translation assays indicate poly-GR and poly-PR peptides inhibit global translation probably by restraining the access of translation factors to mRNA [172]. Overexpression of poly-GR/PR can promote spontaneous assembly of poorly dynamic stress granules and thereafter inhibit global translation [171]. Overexpressing a single translation initiation factor eIF1A alleviates translation repression caused by poly-GR in human cells and rescues DPR-induced toxicity in vivo [174]. More recently, a high-resolution cryogenic electron microscopy (cryo-EM) study reveals that poly-GR/PR binds to the polypeptide exit tunnel of the ribosome and impairs peptidyl transfer which inhibits translation initiation and elongation [176]. This study provides a structure foundation of how poly-GR and poly-PR could interfere with the general translation process. Further studies using in vivo, and especially neuron models, including genome-wide translatome studies, will help reveal more pathophysiological consequences of this defect and its contribution to neurodegeneration.

### FUS

FUS/TLS is an RNA-binding protein that predominantly localizes in the nucleus and shuttles between the nucleus and cytoplasm. FUS protein is involved in many cellular processes including gene expression regulation, DNA repair, alternative splicing, RNA degradation, alternative polyadenylation and translation regulation [177]. Mutations or abnormal aggregation of FUS/TLS have been associated with many neurodegenerative diseases including ALS. Variants in *FUS* account for around 5% of familial ALS [178]. Most of the fALS-related FUS mutations cluster in the N-terminal “prion-like” or low complexity domain, the secondary Arg-Gly-Gly (RGG)-repeat domain, and the nuclear localization signal (NLS) at the C-terminus [179]. FALS-associated FUS mutations alter the biophysical properties and the subcellular localization of FUS proteins. Although the features of FUS inclusions differ in different mutations, tangle-like vs. round shaped, basophilic or not, inclusion formation is recognized as a hallmark of disease [180]. It is worth to mention that FUS inclusions are also observed in sporadic ALS and FTD cases [181].

FUS contains four RNA-binding domains (RBDs): two arginine-glycine-glycine boxes (RGGs), an RNA-recognition motif (RRM), and a zinc finger domain (ZnF) [182]. Stable secondary structures such as stem-loop [183], G-quadruplex [184] or GU-rich motifs such as GGUG [185], GUGGU [182],and a combination of 6 GU-rich (6GU_R_) motifs [186], have been identified as FUS binding sites indicating the divergent role of FUS in RNA metabolism. Mutated FUS undergo liquid-liquid phase separation (LLPS) in cytoplasm and form cytoplasmic FUS condensates [187–189]. The FUS aggregates also contain other RBPs, such as FMRP. After being sequestered, the normal function of FMRP is compromised and the decreased translation of FMRP targets is observed [7, 190, 191]. Proteomic analysis has identified that proteins enriched in translation and RNA quality surveillance pathways are sequestered in FUS inclusions, supporting FUS pathology could induce global decrease of translation [192] (Fig. 3).

FUS is also involved in localized translation (Fig. 3). It has been demonstrated that FUS associates with adenomatous polyposis coli (APC)-containing ribonucleoprotein complexes (APC-RNPs) and facilitates the translation of associating mRNAs especially at protrusive areas of cells [193]. ALS-associated mutant FUS forms cytoplasmic inclusions and disrupts the APC-dependent mRNA localization by impairing kinesin-1 function in both fibroblast cells and primary neuronal cells [194]. In mice expressing human mutant FUS, a 25% reduction in global translation is observed in cultured hippocampal neurons compared with WT mice. More strikingly, the 25% reduction of global protein synthesis is contributed predominantly by the decrease in intra-axonal protein translation [195]. It is shown that the mutant human FUS accumulates at axons, activates integrated stress response, and inhibits localized translation in axons both in vitro and in vivo [195]. This influences the synaptic proteome dynamics and neuron activity.

### TDP43

TAR DNA binding protein 43 kDa (TDP43) is encoded by the TARDBP gene located at chromosomal locus 1p36.22 and is an evolutionarily highly conserved protein belonging to the heterogeneous nuclear ribonucleoprotein (hnRNP) family [196]. TDP43 is ubiquitously expressed in all types of tissues and located predominantly in nucleus although it can shuttle between the nucleus and cytoplasm. TDP43 has been found to be involved in many steps of RNA metabolism, including the regulation of transcription, alternative splicing, mRNA stability and translation [197]. Its dysfunction is associated with several neurodegenerative diseases including ALS [198, 199]. Mutations of TDP43 account for 3% familial ALS cases and 1.5% sporadic ALS cases [200]. But cytoplasmic aggregation and nuclear depletion of TDP43 have been associated with almost all ALS, and have also been found in around half FTD and AD patients [201]. This highlights the importance of understanding the essential roles of TDP43 in neurodegeneration.

TDP43 contains 414 amino acids and can bind to both DNA and RNA. As an hnRNP protein, TDP43 contains all the classical domains of this family including RRM domains, N-terminal domain (NTD), and C-terminal domain (CTD) [196]. TDP43 binds to RNA predominantly depending on the RRM1, although it has been shown that RRM1-RRM2 interaction may influence RNA binding [202]. The glycine-rich CTD is an intrinsically disordered low-complexity domain with the estimation that 36–66% of this region is disordered [196]. The CTD is responsible for the interaction of TDP43 with other proteins [203] and is essential for TDP43 phase separation under physiological conditions [188]. TDP43 is the components of many membrane-less organelles such as stress granule [204] and nuclear stress bodies [205]. The majority of ALS linked mutations are clustered within the CTD [206] which could promote the transition of liquid-like TDP43 droplets to pathological aggregates [188, 207].

TDP43 can bind to mRNA and modulate its translation directly. It has been demonstrated that the translation of genes essential for neurodevelopment and neuron plasticity, such as Rac1 [208, 209], Map1b [209, 210], and GluR1 [209], can be repressed by TDP43. Mechanistically, TDP43 binds to those mRNAs and recruits the CYFIP1-FMRP inhibitory complex via its glycine-rich domain to repress translation [209]. In addition, mutant TDP43 inhibits the translation of hsc70-4 mRNA by RNA sequestration which in turn impairs synaptic vesicle cycling [211]. Paradoxically, TDP43 has also been shown to promote the translation of certain mRNAs. Through ribosome profiling, Camta1, Mig12, and Dennd4a mRNAs are identified as the translational targets of TDP43 [212]. TDP43 enhances the translation of Camta1 and Mig12 mRNAs via binding to their 5’ UTR, yet represses translation of Dennd4a when binding to its 5’ UTR. The translation of Dennd4a mRNA can only be upregulated by TDP43 patient mutant (A315T) through its binding to the 3’ UTR region of Dennd4a mRNA [212]. How different substrates show different translation changes by TDP43 binding needs further exploration.

Global translation can also be influenced by TDP43 (Fig. 3). Cytoplasmic overexpression of TDP43 (TDP43 with NLS deletion) decreases global protein synthesis both in vitro [213] and in vivo [214]. Through translating ribosome affinity purification (TRAP) coupled with RNA-seq, it has been revealed that the ribosome association of numerous mRNAs are altered under TDP-43 proteinopathy [215]. TDP43 may influence global translation through binding to translational machinery. Proteomic study has identified many translation initiation and elongation factors, and ribosomal subunits as TDP43 interacting proteins [216]. Under stress conditions, TDP43 is associated with stalled ribosome and localized to stress granule. This association is dissolved after the removal of stress [217]. Increased cytosolic TDP43 was shown to bind RACK1 on polyribosomes, which contributes to reduced global translation [213]. Additionally, the LLPS of TDP43 is proposed to be important for the translation repression [218]. Axonal TDP43 condensates have been shown to inhibit local protein synthesis of nuclear encoded mitochondrial proteins that are important for neuromuscular junction [219]. Genome-wide RNA stability analysis has demonstrated a profound reduction of ribosomal transcripts in ALS patient-derived cells, including C9ORF72-ALS and sporadic ALS, which can be recapitulated by TDP-43 overexpression [220]. The reduction of RNA transcripts involved in ribosomal biogenesis may further influence global translation and eventually cause cell death in ALS patients. Therefore, TDP43 cytosolic mislocalization and pathological aggregates may influence translation via multiple mechanisms.

### hNRNPA1 and hnRNPA2/B1

Heterogeneous nuclear ribonucleoprotein A1 and A2/B1 are members of the hnRNP family that are involved in many aspects of RNA metabolism including RNA transcription, RNA splicing, RNA trafficking, translation, and RNA degradation [221, 222]. They are both ubiquitously expressed proteins that predominately locate in the nucleus with shuttling between nuclear and cytoplasm [222, 223]. hnRNPA1 and hnRNPA2/B1 contain two RRMs at the N-terminus and a glycine rich domain (also known as prion-like domain (PrLD)) at the C-terminus [221–223]. hnRNPA1 and hnRNPA2/B1 are implicated in many neurodegenerative diseases such as ALS/FTD, AD, HD, SMA and MS (multiple sclerosis) [8, 221, 222]. Mutations in the PrLD domain have been identified in ALS patients [8]. The PrLD domain is important to mediate the LLPS of hnRNPA1 and A2/B1, and the RRMs also contribute to LLPS in the presence of RNA [189, 224, 225]. HnRNPA1 and A2/B1 have been identified as components of stress granules and play important roles in stress response [224, 226]. ALS-associated mutations of hnRNPA1 and A2/B1 change the LLPS properties, increase their accumulation in stress granules and impair the dynamics of stress granule assembly/dissembly [227, 228]. In addition, mutant hnRNPA1 and A2/B1 may also influence the assembly of stress granules by interacting with other proteins such as TDP43 [224, 225], G3BP, TIA1, and FUS directly or indirectly [189, 221].

Both hnRNPA1 and hnRNPA2/B1 have been shown to influence cap-independent translation of target mRNAs. HnRNPA1 has been identified as an IRES trans-activating factor (ITAF) that can bind to IRES sequences and regulate ribosomal entry for cap-independent translation [229]. Human fibroblast growth factor 2 (FGF-2) is identified as the first cap-independent translation target of hnRNPA1. It has been shown that hnRNPA1 binds to the 5’ leader sequence of FGF-2 and stimulates IRES-mediate initiation of the four FGF-2 isoforms but has no effect on cap-dependent initiation [229]. The mRNA of GluA1, a key factor that mediates long-term synaptic plasticity, is demonstrated to be a target of hnRNPA2/B1 and contains an IRES in the 5′UTR. HnRNPA2/B1 binds to an IRES in the 5’UTR of GluA1 mRNA and stimulates the cap-independent translation, which is upregulated upon Brain-derived neurotrophic factor (BDNF) stimulation [230]. hnRNPA1 and hnRNPA2/B1 have also been shown to modulate cap-independent and -dependent translation of several other targets [221, 222]. Further genome-wide study will help decipher how hnRNPA1 and hnRNPA2/B1 modulate the translation of specific targets in neurons, especially in response to stress, neuron activation, growth factor, as well as localized proteome dynamics at synapse.

### ANG

Angiogenin (ANG) is a vertebrate-specific ribonuclease and was first identified and characterized due to its important role in angiogenesis [231]. ANG has relatively weak ribonucleolytic activity compared to other known ribonucleases such as RNase A [231–233], indicating its distinct substrates and functions. ANG shuttles between nucleus and cytoplasm, which is important for its function [234, 235]. It has been demonstrated that ANG is implicated in many physiological processes including angiogenesis, neurogenic, and immune-regulation, and pathological processes such as tumorigenesis and neurodegeneration [231, 236]. ANG loss of function mutations have been identified in AD [237], PD [238], and ALS [238].

ANG plays an important role in translation regulation under physiological and stress conditions. ANG enhances ribosomal RNA synthesis through multiple mechanisms. First, ANG can directly bind to the promoter of rDNA and alter histone modifications, which increases rRNA transcription [239]. Second, ANG cleaves the rRNA promoter-associated RNA thus promotes transcription [240]. Third, ANG may also participate in the maturation of rRNA via cleavage of pre-rRNA [241, 242].

Under stress conditions such as oxidative stress, hypoxia, and starvation, ANG may translocate to cytoplasm and cleave tRNAs which will result in global translation inhibition (Fig. 3). Cleavage at the conserved single-stranded 3’-CCA termini of tRNAs by ANG causes the deactivation of tRNA and repression of translation elongation [243]. ANG also cleaves tRNA at the anticodon loop to produce tRNA-derived, stress-induced small RNAs (tiRNAs) [244–246]. In vitro analysis indicates that tiRNA cooperates with translation silencer YB-1 to prevent translation initiation by displacing eIF4F components [247]. It also has been demonstrated that tiRNA triggers stress granule assembly [248]. Overall, ANG is an important component of stress response that is independent of eIF2α phosphorylation-mediated ISR pathway.

ALS-related ANG variants have been shown to have cytotoxic effects on motor neurons and lack neuroprotective activity under stress [249]. Many mutations influence the ribonuclease activity, the nuclear translocation activity, and the stability of ANG [231, 236]. Given that these properties of ANG are essential to its function in translation regulation, it is reasonable to speculate that translation dysregulation contributes to the pathogenesis of ANG-related ALS.

### TIA1

T-cell intracellular antigen 1 (TIA1) is an RNA binding protein that plays diverse roles in many aspects of RNA metabolism and is ubiquitously expressed [250]. TIA1 contains three RRMs and a PrLD at the C-terminus. The PrLD is enriched in glutamine and asparagine residues, and is essential for stress granule assembly [250, 251]. TIA1 is involved in the regulation of many cellular processes including transcription, splicing, translation, and stress response [250, 251]. TIA1 is a key component of stress granule and often used as the marker protein [252]. TIA1 interacts with many RNAs and proteins and undergoes LLPS to form stress granules under stress conditions [250, 251]. ALS-associated mutations in the PrLD of TIA1 alter the biophysical properties and influence the dynamics of stress granule assembly and disassembly [253, 254] (Fig. 3).

TIA1 has been shown to directly modulate the translation of target mRNAs. TIA1 directly binds to the AU-rich element in the 3’UTR of target mRNAs and represses their translation [255–260].In addition, mRNAs encoding translation factors are among the targets of TIA1. Knockdown of TIA1 increased the relative abundance of ribosomal P0 protein and several canonical initiation factors such as eIF4G, eIF4E, and PABP, which can enhance translation globally [261]. The translation repression by TIA1 has been shown to play important roles in neurodevelopment [262].

## Ribosome-Associated Quality Control (RQC) pathway and its potential link with ALS

Translation elongation can be slowed down under many circumstances, such as the presence of mRNA and rRNA damage, suboptimal codons, mRNA secondary structures, and environmental stresses. This could increase ribosome stalling and cause inefficient ribosome recycling, which is deleterious to cells and needs to be resolved quickly to maintain cell homeostasis. Ribosome-associated protein quality control (RQC) pathway is a dedicated surveillance mechanism that is used to monitor ribosome stalling. During RQC, stalled ribosomes can be detected and dissociated into subunits by specific factors for recycling [164, 263]. NEMF (Nuclear Export Mediator Factor) and LTN1 (Listerin E3 ubiquitin protein ligase 1) are two key components of ribosome-associated quality control (RQC) pathway that is important in maintaining proteostasis [164, 263] (Fig. 3). NEMF senses large ribosomal subunits obstructed with peptidyl-tRNA and triggers polyubiquitination of the nascent chains by recruiting LTN1 E3 ligase and stabilizing the interaction between LTN1 and 60 S subunits [264]. NEMF can add C-terminal tails to the nascent chains independent of mRNA template and the small ribosomal subunits. This facilitates the exposure of lysine residues buried in ribosomes for ubiquitination of the nascent peptides, which are further degraded by the proteasome [164, 263]. Although no mutation in RQC-related genes has been identified in ALS to date, accumulating evidence has suggested that RQC pathway disruption is implicated in neurodegeneration. As mentioned in the previous section, arginine-rich DPRs may cause ribosome stalling and activate RQC pathway. In addition, mice carrying mutations in RQC-related genes show neurological and motor dysfunction.

Through ENU-mediated random mutagenesis, mice with mutations in both LTN1 [265] and NEMF [266] show ALS-like phenotypes. In mice with LTN1 mutation, motor neuron degeneration (including the loss of motor neurons and the reduction in the number and diameter of the axons) in the spinal cord at the terminal stage is observed [265]. Homozygous NEMF mutations (R86S and R487G) result in progressive motor phenotypes including abnormal gait and progressive axonal degeneration. The phenotypic severity of mice is correlated with the reduction in the C-terminal tailing activity of NEMF [266]. NEMF mutations have been identified in several families with neuromuscular disease [266, 267]. Those studies indicate that dysregulation of translation elongation and RQC pathways are important for motor neuron degeneration. Whether there are pathological features or genetic risk factors involved in sporadic ALS is worth further investigation.

## Conclusion and future perspectives

Much progress has been made over the past few years in the etiology of ALS including the discovery of a handful of RBPs whose mutations and pathology have been strongly linked with ALS. As mentioned above, under physiological conditions those proteins have been demonstrated to either play essential roles or carry regulatory functions in RNA translation and their ALS-linked mutations or pathology may disrupt their normal function in translational control. The fact that many ALS-associated RBPs can influence the function of other RBPs by regulating their expression and/or activity adds another layer of complexity in translation dysregulation. ISR is dysregulated in many neurological diseases including ALS. Many RBPs that are linked with ALS are components of stress granules. ALS-linked mutations in those RBPs can influence the dynamics of stress granule assembly and disassembly which contributes to ISR activation. Thus, the RBP dysfunction may also induce global translation dysregulation besides the subsets of RBP-specific targets. Depending on the individual functions of different RBPs, the translation perturbation could contribute to neurodegeneration via different mechanisms.

There has been a lot of progress understanding the repeat associated translation of the C9ORF72 repeat expansion. Previous studies identified the cis-acting elements, trans-acting factors, as well as cell signaling pathways that can modulate the repeat translation initiation efficiency. The potential RNA species that is subjected to the nuclear export and translation in the splicing context was also revealed. These studies on C9ORF72 repeat RNA translation mechanisms also shed light on other repeat expansion diseases. There are also many questions remain to be answered. The translation of the antisense repeats could be less complicated, as there is no splicing involved. The antisense transcript likely contains the 5’ cap and is more efficiently exported to cytoplasm for translation. It is noted that there are AUG start codons in the GP and PR frames, 194 and 273 nt upstream of the antisense repeat expansion respectively [268]. If the transcription of the antisense starts before the start codons, the poly-GP/PR should be produced by the canonical translation. Therefore, mapping the transcription start site of the antisense strand is critical for understanding the translation mechanism. However, the production of poly-PA still requires the repeat-associated translation, and the antisense repeat sequences and structures might still influence poly-GP and poly-PR even if they initiate from AUG start codon. It is also likely that specific RBPs bound on the antisense repeat RNA could modulate the translation outcome. Although DPRs translated from all reading frames have been detected in patient by various methods including IHC and ELISA, the relative abundance of different DPRs remains unresolved to date due to technical limitations. Deciphering the relative abundance of different DPRs is of particular importance in understanding the disease etiology as the cytotoxicity of different DPRs varies significantly. Thus, development of advanced techniques that enable the comparison of different DPR levels, particularly in patients, are highly valuable to the field.

Although many translation initiation factors and RBPs have been reported to regulate the GGGGCC repeat translation, these factors were usually identified using non-neuronal cell types. It will be interesting to explore whether there are neuron-specific factors modulating the repeat translation and whether there are cell type differences of DPR production. Furthermore, as repeat RNA can be found in synapse [269], whether there is localized RAN translation requires further investigation. Besides the focus on the translation initiation of the repeat RNA, more efforts need to be extended to translation elongation, frameshifting, and ribosome quality control pathways. It is known that the ribosome translocation is not uniform and there is higher tendency of stalling on specific amino acid sequences. As the repeat expansion encodes different repetitive peptide sequences, it is likely the elongation through the different reading frames has different speed. Additionally, it is also interesting to determine whether the slowed elongation and/or the secondary structure of the repeat RNA can enhance the frameshifting events between the different reading frames. How much poly-GP and poly-GR are generated from the in-frame initiation or frameshifting from the GA frame, and how prevalent the chimeric DPRs can be produced from frameshifting need to be determined. Furthermore, if the ribosome translocation is slowed down, this will potentially increase the chance of ribosome collision, especially if different frames have different elongation speeds. Whether RQC pathways will be activated and how it influences the DPR production requires further study.

Furthermore, it is intriguing to understand the pathophysiological role of translational defects caused by ALS-linked genes, including RBPs and poly-GR/PR. Translatome studies could help reveal the substrate specificity in vivo, such as whether certain mRNA and amino acid sequences are preferentially influenced, and any different impact on localized translation in synapse, etc. Recent studies also suggest heterogeneity in the translational machinery in different cell types and developmental stages [270]. Additionally, different expression levels of components in the ribosome quality control pathways might determine the sensitivity of cells to the insults of translation errors and the activation of downstream stress signaling. Therefore, it is important to dissect the translational dysregulation triggered by the mutant genes in neurons and decipher how this contributes to neurodegeneration phenotypes.

Taken together, increasing evidence suggests that the delicate RNA translation regulation plays important roles in neuronal homeostasis. Accumulating studies indicate that dysregulation of RNA translation could preferentially result in neurological and neurodegenerative diseases. Recent development of novel technology and experimental approaches allows advances in understanding the various molecular mechanism of translation regulation at different steps and at the genome-wide level. Despite the progress on the fundamental process in all cells, how the pathways and translatome are fine-tuned in neurons for the highly specialized morphology and function is relatively less studied. Moving forward, it is important to dissect translational changes in specific neuronal and glial cell types and at specific pathological conditions. For example, a cascade of cell type-specific and age-dependent translatome changes caused by mutant SOD1 in mouse spinal cord was identified using the TRAP coupled RNA-seq approach [271]. Similar techniques could be applied to other models with RBP defects. Future development of spatial translatomics and single-cell translatomics [272] will also advance the understanding of localized and cell type specific translation dysregulation in human patient postmortem tissues directly. Furthermore, it is critical to study the interactions of different RBPs in translation regulation, distinguish the molecular mechanisms of direct and indirect influences on translation. It is also intriguing to develop pharmacological strategies to rescue translation dysregulation and assess their therapeutic values.

## Abbreviations

ALS: Amyotrophic Lateral Sclerosis
FTD: Frontotemporal Dementia
RBP: RNA binding protein
ORF: open reading frame
uORF: upstream ORF
eIF: eukaryotic initiation factor
eEF: eukaryotic elongation factor
eRF: eukaryotic release factor
PIC: pre-initiation complex
PTC: peptidyl-transferase center
AARS: aminoacyl-tRNA synthetase
IRES: internal ribosome entry site
ITAF: IRES-trans activating factor
RAN translation: Repeat-associated non-AUG translation
DPR protein: Dipeptide repeat protein
ISR: Integrated stress response
NMD: nonsense mediated decay
AD: Alzheimer’s Disease
PD: Parkinson’s Disease
HD: Huntington’s Disease
DM: Myotonic Dystrophy
FXTAS: Fragile X-associated tremor/ataxia syndrome
FXS: fragile X syndrome
SCA: Spinocerebellar Ataxia
CMT: Charcot-Marie-Tooth disease
SMA: spinal muscular atrophy
RQC: ribosome-associated quality control
smFISH: single-molecule Fluorescence in Situ Hybridization
SINAPS: single-molecule imaging of nascent peptides
LLPS: liquid-liquid phase separation
PrLD: prion-like domain
NLS: nuclear localization signal

## References

[1] Tandan R, Bradley WG (1985). Amyotrophic lateral sclerosis: part 1. Clinical features, pathology, and ethical issues in management. Annals of Neurology: Official Journal of the American Neurological Association and the Child Neurology Society.

[2] Mehta P et al. Prevalence of amyotrophic lateral sclerosis (ALS), United States, 2016. Amyotroph Lateral Scler Frontotemporal Degeneration, 1–6 (2021). 10.1080/21678421.2021.1949021 34423697

[3] Gros-Louis F, Gaspar C, Rouleau GA (2006). Genetics of familial and sporadic amyotrophic lateral sclerosis. Biochim et Biophys Acta (BBA)-Molecular Basis Disease.

[4] Hershey JW, Sonenberg N, Mathews MB (2019). Principles of translational control. Cold Spring Harb Perspect Biol.

[5] Hentze MW, Castello A, Schwarzl T, Preiss T (2018). A brave new world of RNA-binding proteins. Nat Rev Mol Cell Biol.

[6] Gerstberger S, Hafner M, Tuschl T (2014). A census of human RNA-binding proteins. Nat Rev Genet.

[7] Thelen MP, Kye MJ (2020). The role of RNA binding proteins for local mRNA translation: implications in neurological disorders. Front Mol Biosci.

[8] Kapeli K, Martinez FJ, Yeo GW (2017). Genetic mutations in RNA-binding proteins and their roles in ALS. Hum Genet.

[9] Hetz C, Zhang K, Kaufman RJ (2020). Mechanisms, regulation and functions of the unfolded protein response. Nat Rev Mol Cell Biol.

[10] Wolozin B, Ivanov P (2019). Stress granules and neurodegeneration. Nat Rev Neurosci.

[11] Gao F-B, Richter JD, Cleveland DW (2017). Rethinking unconventional translation in neurodegeneration. Cell.

[12] Mori K (2013). The C9orf72 GGGGCC repeat is translated into aggregating dipeptide-repeat proteins in FTLD/ALS. Science.

[13] Ash PE (2013). Unconventional translation of C9ORF72 GGGGCC expansion generates insoluble polypeptides specific to c9FTD/ALS. Neuron.

[14] Zu T et al. RAN proteins and RNA foci from antisense transcripts in C9ORF72 ALS and frontotemporal dementia. *Proceedings of the National Academy of Sciences* 110, E4968-E4977 (2013). 10.1073/pnas.1315438110 PMC3870665 24248382

[15] Dever TE, Green R (2012). The elongation, termination, and recycling phases of translation in eukaryotes. Cold Spring Harb Perspect Biol.

[16] Jackson RJ, Hellen CU, Pestova TV (2010). The mechanism of eukaryotic translation initiation and principles of its regulation. Nat Rev Mol Cell Biol.

[17] Saba JA, Liakath-Ali K, Green R, Watt FM (2021). Translational control of stem cell function. Nat Rev Mol Cell Biol.

[18] Dever TE, Dinman JD, Green R (2018). Translation elongation and recoding in eukaryotes. Cold Spring Harb Perspect Biol.

[19] Gomez MAR, Ibba M (2020). Aminoacyl-tRNA synthetases. RNA.

[20] Song H (2000). The crystal structure of human eukaryotic release factor eRF1—mechanism of stop codon recognition and peptidyl-tRNA hydrolysis. Cell.

[21] Hellen CU (2018). Translation termination and ribosome recycling in eukaryotes. Cold Spring Harb Perspect Biol.

[22] Buttgereit F, Brand MD (1995). A hierarchy of atp-consuming processes in mammalian-cells. Biochem J.

[23] Russell JB, Cook GM (1995). Energetics of bacterial-growth - balance of anabolic and catabolic reactions. Microbiol Rev.

[24] Aoyagi Y, Tasaki I, Okumura J-i, Muramatsu T (1988). Energy cost of whole-body protein synthesis measured in vivo in chicks. Comp Biochem Physiol Comp Physiol.

[25] Holt CE, Martin KC, Schuman EM (2019). Local translation in neurons: visualization and function. Nat Struct Mol Biol.

[26] Buxbaum AR, Haimovich G, Singer RH (2015). In the right place at the right time: visualizing and understanding mRNA localization. Nat Rev Mol Cell Biol.

[27] Wu B, Eliscovich C, Yoon YJ, Singer RH (2016). Translation dynamics of single mRNAs in live cells and neurons. Science.

[28] Amorim IS, Lach G, Gkogkas CG. The role of the eukaryotic translation initiation factor 4E (eIF4E) in Neuropsychiatric Disorders. Front Genet 9 (2018). 10.3389/fgene.2018.00561 PMC6265315 30532767

[29] Napoli I (2008). The fragile X syndrome protein represses activity-dependent translation through CYFIP1, a new 4E-BP. Cell.

[30] Richter JD, Sonenberg N (2005). Regulation of cap-dependent translation by eIF4E inhibitory proteins. Nature.

[31] Krishnamoorthy T, Pavitt GD, Zhang F, Dever TE, Hinnebusch AG (2001). Tight binding of the phosphorylated alpha subunit of initiation factor 2 (eIF2 alpha) to the regulatory subunits of guanine nucleotide exchange factor eIF2B is required for inhibition of translation initiation. Mol Cell Biol.

[32] Bogorad AM, Lin KY, Marintchev A (2018). eIF2B mechanisms of action and regulation: a thermodynamic view. Biochemistry-Us.

[33] Garcia M (2006). Impact of protein kinase PKR in cell biology: from antiviral to antiproliferative action. Microbiol Mol Biol Rev.

[34] Li G, Scull C, Ozcan L, Tabas I (2010). NADPH oxidase links endoplasmic reticulum stress, oxidative stress, and PKR activation to induce apoptosis. J Cell Biol.

[35] Udumula MP (2017). High glucose impairs insulin signaling via activation of PKR pathway in L6 muscle cells. Biochem Bioph Res Co.

[36] Nakamura T (2015). A critical role for PKR Complexes with TRBP in Immunometabolic Regulation and eIF2 alpha phosphorylation in obesity. Cell Rep.

[37] Donnelly N, Gorman AM, Gupta S, Samali A (2013). The eIF2α kinases: their structures and functions. Cell Mol Life Sci.

[38] Deng J (2002). Activation of GCN2 in UV-irradiated cells inhibits translation. Curr Biol.

[39] de la Cadena SC, Hernandez-Fonseca K, Camacho-Arroyo I, Massieu L (2014). Glucose deprivation induces reticulum stress by the PERK pathway and caspase-7-and calpain-mediated caspase-12 activation. Apoptosis.

[40] Girardin SE, Cuziol C, Philpott DJ, Arnoult D (2021). The eIF2α kinase HRI in innate immunity, proteostasis, and mitochondrial stress. FEBS J.

[41] Bond S, Lopez-Lloreda C, Gannon PJ, Akay-Espinoza C, Jordan-Sciutto KL (2020). The integrated stress response and phosphorylated eukaryotic initiation factor 2α in neurodegeneration. J Neuropathology Experimental Neurol.

[42] Hetz C, Saxena S (2017). ER stress and the unfolded protein response in neurodegeneration. Nat Reviews Neurol.

[43] Dugger BN, Dickson DW (2017). Pathology of neurodegenerative diseases. Cold Spring Harb Perspect Biol.

[44] Moreno JA (2012). Sustained translational repression by eIF2α-P mediates prion neurodegeneration. Nature.

[45] Derisbourg MJ, Hartman MD, Denzel MS (2021). Modulating the integrated stress response to slow aging and ameliorate age-related pathology. Nat aging.

[46] Sidrauski C, McGeachy AM, Ingolia NT, Walter P (2015). The small molecule ISRIB reverses the effects of eIF2α phosphorylation on translation and stress granule assembly. elife.

[47] Oliveira MM et al. Correction of eIF2-dependent defects in brain protein synthesis, synaptic plasticity, and memory in mouse models of Alzheimer’s disease. Sci Signal 14 (2021). 10.1126/scisignal.abc5429 PMC8317334 33531382

[48] Krukowski K (2020). Small molecule cognitive enhancer reverses age-related memory decline in mice. Elife.

[49] Silva RM (2005). CHOP/GADD153 is a mediator of apoptotic death in substantia nigra dopamine neurons in an in vivo neurotoxin model of parkinsonism. J Neurochem.

[50] Kim H-J (2014). Therapeutic modulation of eIF2α phosphorylation rescues TDP-43 toxicity in amyotrophic lateral sclerosis disease models. Nat Genet.

[51] Wang L, Popko B, Roos RP (2014). An enhanced integrated stress response ameliorates mutant SOD1-induced ALS. Hum Mol Genet.

[52] Jiang H-Q (2014). Guanabenz delays the onset of disease symptoms, extends lifespan, improves motor performance and attenuates motor neuron loss in the SOD1 G93A mouse model of amyotrophic lateral sclerosis. Neuroscience.

[53] Saxena S, Cabuy E, Caroni P (2009). A role for motoneuron subtype–selective ER stress in disease manifestations of FALS mice. Nat Neurosci.

[54] Colla E (2012). Accumulation of toxic α-synuclein oligomer within endoplasmic reticulum occurs in α-synucleinopathy in vivo. J Neurosci.

[55] Krzyzosiak A (2018). Target-based discovery of an inhibitor of the regulatory phosphatase PPP1R15B. Cell.

[56] Van Der Knaap MS (2002). Mutations in each of the five subunits of translation initiation factor eIF2B can cause leukoencephalopathy with vanishing white matter. Annals of Neurology: Official Journal of the American Neurological Association and the Child Neurology Society.

[57] Gat-Viks I, Geiger T, Barbi M, Raini G, Elroy‐Stein O (2015). Proteomics‐level analysis of myelin formation and regeneration in a mouse model for vanishing white matter disease. J Neurochem.

[58] Moon SL, Parker R (2018). EIF2B2 mutations in vanishing white matter disease hypersuppress translation and delay recovery during the integrated stress response. RNA.

[59] Van Der Voorn JP (2005). The unfolded protein response in vanishing white matter disease. J Neuropathology Experimental Neurol.

[60] Lin KW, Yakymovych I, Jia M, Yakymovych M, Souchelnytskyi S (2010). Phosphorylation of eEF1A1 at Ser300 by T beta R-I results in inhibition of mRNA translation. Curr Biol.

[61] Qin SY, Ye L, Zheng YS, Gao J (2021). Cytosolic PINK1 orchestrates protein translation during proteasomal stress by phosphorylating the translation elongation factor eEF1A1. Febs Lett.

[62] Jakobsson ME, Malecki J, Falnes PO (2018). Regulation of eukaryotic elongation factor 1 alpha (eEF1A) by dynamic lysine methylation. Rna Biol.

[63] Carlberg U, Nilsson A, Nygard O (1990). Functional-Properties of phosphorylated elongation Factor-Ii. Eur J Biochem.

[64] Celis JE, Madsen P, Ryazanov AG (1990). Increased phosphorylation of elongation Factor-Ii during mitosis in Transformed Human Amnion cells correlates with a decreased rate of protein-synthesis. P Natl Acad Sci USA.

[65] Lee S, Wolfraim LA, Wang E (1993). Differential expression of S1 and elongation factor-1 alpha during rat development. J Biol Chem.

[66] Chambers DM, Peters J, Abbott CM (1998). The lethal mutation of the mouse wasted (wst) is a deletion that abolishes expression of a tissue-specific isoform of translation elongation factor 1α, encoded by the Eef1a2 gene. Proc Natl Acad Sci.

[67] Newbery HJ (2005). Progressive loss of motor neuron function in wasted mice: effects of a spontaneous null mutation in the gene for the eEF1A2 translation factor. J Neuropathology Experimental Neurol.

[68] Sandbaken MG, Culbertson MR (1988). Mutations in elongation factor EF-1 alpha affect the frequency of frameshifting and amino acid misincorporation in Saccharomyces cerevisiae. Genetics.

[69] De Ligt J (2012). Diagnostic exome sequencing in persons with severe intellectual disability. N Engl J Med.

[70] Lam WW (2016). Novel de novo EEF 1A2 missense mutations causing epilepsy and intellectual disability. Mol Genet Genom Med.

[71] Nakajima J (2015). D e novo EEF1A2 mutations in patients with characteristic facial features, intellectual disability, autistic behaviors and epilepsy. Clin Genet.

[72] Kaur S (2019). Whole exome sequencing reveals a de novo missense variant in EEF1A2 in a Rett syndrome-like patient. Clin Case Rep.

[73] Wei N, Zhang Q, Yang X-L (2019). Neurodegenerative Charcot–Marie–Tooth disease as a case study to decipher novel functions of aminoacyl-tRNA synthetases. J Biol Chem.

[74] Niehues S (2015). Impaired protein translation in Drosophila models for Charcot–Marie–Tooth neuropathy caused by mutant tRNA synthetases. Nat Commun.

[75] Storkebaum E (2009). Dominant mutations in the tyrosyl-tRNA synthetase gene recapitulate in Drosophila features of human Charcot–Marie–Tooth neuropathy. Proc Natl Acad Sci.

[76] Zuko A (2021). tRNA overexpression rescues peripheral neuropathy caused by mutations in tRNA synthetase. Science.

[77] Mendonsa S, von Kuegelgen N, Bujanic L, Chekulaeva M (2021). Charcot–Marie–Tooth mutation in glycyl-tRNA synthetase stalls ribosomes in a pre-accommodation state and activates integrated stress response. Nucleic Acids Res.

[78] Ishimura R, Nagy G, Dotu I, Chuang JH, Ackerman SL (2016). Activation of GCN2 kinase by ribosome stalling links translation elongation with translation initiation. Elife.

[79] Spaulding EL (2021). The integrated stress response contributes to tRNA synthetase–associated peripheral neuropathy. Science.

[80] Mellado W, Willis DE (2021). Stressing out translation. Science.

[81] Ishimura R (2014). Ribosome stalling induced by mutation of a CNS-specific tRNA causes neurodegeneration. Science.

[82] Gavis ER, Lehmann R (1992). Localization of nanos RNA controls embryonic polarity. Cell.

[83] Paquin N, Chartrand P (2008). Local regulation of mRNA translation: new insights from the bud. Trends Cell Biol.

[84] Katz ZB (2012). β-Actin mRNA compartmentalization enhances focal adhesion stability and directs cell migration. Genes Dev.

[85] Khalil B, Morderer D, Price PL, Liu F, Rossoll W (2018). mRNP assembly, axonal transport, and local translation in neurodegenerative diseases. Brain Res.

[86] Gamarra M, De la Cruz A, Blanco-Urrejola M, Baleriola J. Local translation in nervous system pathologies. Front Integr Nuerosci, 16 (2021). 10.3389/fnint.2021.689208 PMC8279726 34276318

[87] Cajigas IJ (2012). The local transcriptome in the synaptic neuropil revealed by deep sequencing and high-resolution imaging. Neuron.

[88] Kar AN, Lee SJ, Twiss JL (2018). Expanding axonal transcriptome brings new functions for axonally synthesized proteins in health and disease. The Neuroscientist.

[89] Shigeoka T (2016). Dynamic axonal translation in developing and mature visual circuits. Cell.

[90] Guillaud L, El-Agamy SE, Otsuki M, Terenzio M (2020). Anterograde axonal transport in neuronal homeostasis and disease. Front Mol Neurosci.

[91] Hirokawa N, Niwa S, Tanaka Y (2010). Molecular motors in neurons: transport mechanisms and roles in brain function, development, and disease. Neuron.

[92] Müntjes K, Devan SK, Reichert AS, Feldbrügge M (2021). Linking transport and translation of mRNAs with endosomes and mitochondria. EMBO Rep.

[93] Liao Y-C (2019). RNA granules hitchhike on lysosomes for long-distance transport, using annexin A11 as a molecular tether. Cell.

[94] Chaytow H, Huang Y-T, Gillingwater TH, Faller KM (2018). The role of survival motor neuron protein (SMN) in protein homeostasis. Cell Mol Life Sci.

[95] Donlin-Asp PG (2017). The survival of motor neuron protein acts as a molecular chaperone for mRNP assembly. Cell Rep.

[96] Fallini C (2011). The survival of motor neuron (SMN) protein interacts with the mRNA-binding protein HuD and regulates localization of poly (A) mRNA in primary motor neuron axons. J Neurosci.

[97] Saal L, Briese M, Kneitz S, Glinka M, Sendtner M (2014). Subcellular transcriptome alterations in a cell culture model of spinal muscular atrophy point to widespread defects in axonal growth and presynaptic differentiation. RNA.

[98] Nicolas A (2018). Genome-wide analyses identify KIF5A as a novel ALS gene. Neuron.

[99] Hirokawa N (2006). mRNA transport in dendrites: RNA granules, motors, and tracks. J Neurosci.

[100] Kanai Y, Dohmae N, Hirokawa N (2004). Kinesin transports RNA: isolation and characterization of an RNA-transporting granule. Neuron.

[101] Sznajder ŁJ, Swanson MS (2019). Short tandem repeat expansions and RNA-mediated pathogenesis in myotonic dystrophy. Int J Mol Sci.

[102] Cleary JD, Pattamatta A, Ranum LP (2018). Repeat-associated non-ATG (RAN) translation. J Biol Chem.

[103] Zu T et al. Non-ATG–initiated translation directed by microsatellite expansions. *Proceedings of the National Academy of Sciences* 108, 260–265 (2011). 10.1073/pnas.1013343108 PMC3017129 21173221

[104] Banez-Coronel M, Ranum LP (2019). Repeat-associated non-AUG (RAN) translation: insights from pathology. Lab Invest.

[105] Todd PK (2013). CGG repeat-associated translation mediates neurodegeneration in fragile X tremor ataxia syndrome. Neuron.

[106] Zu T (2017). RAN translation regulated by muscleblind proteins in myotonic dystrophy type 2. Neuron.

[107] Ishiguro T (2017). Regulatory role of RNA chaperone TDP-43 for RNA misfolding and repeat-associated translation in SCA31. Neuron.

[108] McEachin ZT (2020). Chimeric peptide species contribute to divergent dipeptide repeat pathology in c9ALS/FTD and SCA36. Neuron.

[109] Bañez-Coronel M (2015). RAN translation in Huntington disease. Neuron.

[110] Mizielinska S (2013). C9orf72 frontotemporal lobar degeneration is characterised by frequent neuronal sense and antisense RNA foci. Acta Neuropathol.

[111] Gendron TF (2013). Antisense transcripts of the expanded C9ORF72 hexanucleotide repeat form nuclear RNA foci and undergo repeat-associated non-ATG translation in c9FTD/ALS. Acta Neuropathol.

[112] Lagier-Tourenne C (2013). Targeted degradation of sense and antisense C9orf72 RNA foci as therapy for ALS and frontotemporal degeneration. Proc Natl Acad Sci.

[113] DeJesus-Hernandez M (2011). Expanded GGGGCC hexanucleotide repeat in noncoding region of C9ORF72 causes chromosome 9p-linked FTD and ALS. Neuron.

[114] Renton AE (2011). A hexanucleotide repeat expansion in C9ORF72 is the cause of chromosome 9p21-linked ALS-FTD. Neuron.

[115] Mori K (2013). Bidirectional transcripts of the expanded C9orf72 hexanucleotide repeat are translated into aggregating dipeptide repeat proteins. Acta Neuropathol.

[116] Freibaum BD, Taylor JP (2017). The role of dipeptide repeats in C9ORF72-related ALS-FTD. Front Mol Neurosci.

[117] Casolari JM, Silver PA (2004). Guardian at the gate: preventing unspliced pre-mRNA export. Trends Cell Biol.

[118] Hesselberth JR (2013). Lives that introns lead after splicing. Wiley Interdisciplinary Reviews: RNA.

[119] Wang S (2021). Nuclear export and translation of circular repeat-containing intronic RNA in C9ORF72-ALS/FTD. Nat Commun.

[120] Niblock M (2016). Retention of hexanucleotide repeat-containing intron in C9orf72 mRNA: implications for the pathogenesis of ALS/FTD. Acta Neuropathol Commun.

[121] Sznajder ŁJ et al. Intron retention induced by microsatellite expansions as a disease biomarker. *Proceedings of the National Academy of Sciences* 115, 4234–4239 (2018). 10.1073/pnas.1716617115 PMC5910826 29610297

[122] Mori K (2013). hnRNP A3 binds to GGGGCC repeats and is a constituent of p62-positive/TDP43-negative inclusions in the hippocampus of patients with C9orf72 mutations. Acta Neuropathol.

[123] Cooper-Knock J (2014). Sequestration of multiple RNA recognition motif-containing proteins by C9orf72 repeat expansions. Brain.

[124] Wickramasinghe VO, Laskey RA (2015). Control of mammalian gene expression by selective mRNA export. Nat Rev Mol Cell Biol.

[125] Cheng W (2019). CRISPR-Cas9 screens identify the RNA helicase DDX3X as a repressor of C9ORF72 (GGGGCC) n repeat-associated non-AUG translation. Neuron.

[126] Hautbergue GM (2017). SRSF1-dependent nuclear export inhibition of C9ORF72 repeat transcripts prevents neurodegeneration and associated motor deficits. Nat Commun.

[127] Malik I (2021). SRSF protein kinase 1 modulates RAN translation and suppresses CGG repeat toxicity. EMBO Mol Med.

[128] Shatsky IN, Terenin IM, Smirnova VV, Andreev DE (2018). Cap-Independent translation: what’s in a name?. Trends Biochem Sci.

[129] Kearse MG (2016). CGG Repeat-Associated Non-AUG Translation utilizes a Cap-Dependent scanning mechanism of initiation to produce toxic proteins. Mol Cell.

[130] Cheng W (2018). C9ORF72 GGGGCC repeat-associated non-AUG translation is upregulated by stress through eIF2α phosphorylation. Nat Commun.

[131] Green KM et al. RAN translation at C9orf72-associated repeat expansions is selectively enhanced by the integrated stress response. Nat Commun 8 (2017). 10.1038/s41467-017-02200-0 PMC5722904 29222490

[132] Sonobe Y (2018). Translation of dipeptide repeat proteins from the C9ORF72 expanded repeat is associated with cellular stress. Neurobiol Dis.

[133] van‘t Spijker HM (2022). Ribosome profiling reveals novel regulation of C9ORF72 GGGGCC repeat-containing RNA translation. RNA.

[134] Tabet R et al. CUG initiation and frameshifting enable production of dipeptide repeat proteins from ALS/FTD C9ORF72 transcripts. Nat Commun 9 (2018). 10.1038/s41467-017-02643-5 PMC5764992 29323119

[135] Hertz MI, Landry DM, Willis AE, Luo G, Thompson SR (2013). Ribosomal protein S25 dependency reveals a common mechanism for diverse internal ribosome entry sites and ribosome shunting. Mol Cell Biol.

[136] Nishiyama T, Yamamoto H, Uchiumi T, Nakashima N (2007). Eukaryotic ribosomal protein RPS25 interacts with the conserved loop region in a dicistroviral intergenic internal ribosome entry site. Nucleic Acids Res.

[137] Yamada SB (2019). RPS25 is required for efficient RAN translation of C9orf72 and other neurodegenerative disease-associated nucleotide repeats. Nat Neurosci.

[138] Ayhan F et al. SCA8 RAN polySer protein preferentially accumulates in white matter regions and is regulated by eIF3F. Embo J 37 (2018). 10.15252/embj.201899023 PMC6166133 30206144

[139] Sonobe Y (2021). A C. elegans model of C9orf72-associated ALS/FTD uncovers a conserved role for eIF2D in RAN translation. Nat Commun.

[140] Green KM, Miller SL, Malik I, Todd PK. Non-canonical initiation factors modulate repeat-associated non-AUG translation. Hum Mol Genet (2022). 10.1093/hmg/ddac021 PMC9618161 35220421

[141] Goodman LD (2019). eIF4B and eIF4H mediate GR production from expanded G4C2 in a Drosophila model for C9orf72-associated ALS. Acta Neuropathol Commun.

[142] Fratta P (2012). C9orf72 hexanucleotide repeat associated with amyotrophic lateral sclerosis and frontotemporal dementia forms RNA G-quadruplexes. Sci Rep.

[143] Su Z (2014). Discovery of a biomarker and lead small molecules to target r (GGGGCC)-associated defects in c9FTD/ALS. Neuron.

[144] Liu H (2021). A helicase unwinds hexanucleotide repeat RNA G-quadruplexes and facilitates repeat-associated non-AUG translation. J Am Chem Soc.

[145] Tseng Y-J et al. The RNA helicase DHX36–G4R1 modulates C9orf72 GGGGCC hexanucleotide repeat–associated translation. J Biol Chem 297 (2021). 10.1016/j.jbc.2021.100914 PMC8326427 34174288

[146] Costa-Mattioli M, Walter P (2020). The integrated stress response: from mechanism to disease. Science.

[147] Westergard T (2019). Repeat-associated non‐AUG translation in C9orf72‐ALS/FTD is driven by neuronal excitation and stress. EMBO Mol Med.

[148] Axten JM (2012). Discovery of 7-methyl-5-(1-{[3-(trifluoromethyl) phenyl] acetyl}-2, 3-dihydro-1 H-indol-5-yl)-7 H-pyrrolo [2, 3-d] pyrimidin-4-amine (GSK2606414), a potent and selective first-in-class inhibitor of protein kinase R (PKR)-like endoplasmic reticulum kinase (PERK). J Med Chem.

[149] Szebényi K (2021). Human ALS/FTD brain organoid slice cultures display distinct early astrocyte and targetable neuronal pathology. Nat Neurosci.

[150] Zu T et al. Metformin inhibits RAN translation through PKR pathway and mitigates disease in C9orf72 ALS/FTD mice. *Proceedings of the National Academy of Sciences* 117, 18591–18599 (2020). 10.1073/pnas.2005748117 PMC7414156 32690681

[151] Donnelly CJ (2013). RNA toxicity from the ALS/FTD C9ORF72 expansion is mitigated by antisense intervention. Neuron.

[152] Wainger BJ, Cudkowicz ME (2015). Cortical hyperexcitability in amyotrophic lateral sclerosis: C9orf72 repeats. JAMA Neurol.

[153] Wainger BJ (2014). Intrinsic membrane hyperexcitability of amyotrophic lateral sclerosis patient-derived motor neurons. Cell Rep.

[154] Penn WD, Harrington HR, Schlebach JP, Mukhopadhyay S (2020). Regulators of viral frameshifting: more than RNA influences translation events. Annual Rev Virol.

[155] Celik A, He F, Jacobson A (2017). NMD monitors translational fidelity 24/7. Curr Genet.

[156] Gaspar C (2000). CAG tract of MJD-1 may be prone to frameshifts causing polyalanine accumulation. Hum Mol Genet.

[157] Girstmair H (2013). Depletion of cognate charged transfer RNA causes translational frameshifting within the expanded CAG stretch in huntingtin. Cell Rep.

[158] Wright SE (2022). CGG repeats trigger translational frameshifts that generate aggregation-prone chimeric proteins. Nucleic Acids Res.

[159] Ebbert MT (2018). Long-read sequencing across the C9orf72 ‘GGGGCC’repeat expansion: implications for clinical use and genetic discovery efforts in human disease. Mol neurodegeneration.

[160] Almeida S (2019). Production of poly (GA) in C9ORF72 patient motor neurons derived from induced pluripotent stem cells. Acta Neuropathol.

[161] Lyon K, Aguilera LU, Morisaki T, Munsky B, Stasevich TJ (2019). Live-cell single RNA imaging reveals bursts of translational frameshifting. Mol Cell.

[162] Quax TE, Claassens NJ, Söll D, van der Oost J (2015). Codon bias as a means to fine-tune gene expression. Mol Cell.

[163] Radwan M (2020). Arginine in C9ORF72 dipolypeptides mediates promiscuous proteome binding and multiple modes of toxicity. Mol Cell Proteomics.

[164] Joazeiro CA (2019). Mechanisms and functions of ribosome-associated protein quality control. Nat Rev Mol Cell Biol.

[165] Park J (2021). ZNF598 co-translationally titrates poly (GR) protein implicated in the pathogenesis of C9ORF72-associated ALS/FTD. Nucleic Acids Res.

[166] Li S et al. Quality-control mechanisms targeting translationally stalled and C-terminally extended poly (GR) associated with ALS/FTD. *Proceedings of the National Academy of Sciences* 117, 25104–25115 (2020). 10.1073/pnas.2005506117 PMC7547246 32958650

[167] Kriachkov V et al. Arginine-rich C9ORF72 ALS proteins stall ribosomes in a manner distinct from a canonical ribosome-associated quality control substrate. J Biol Chem 299 (2023). 10.1016/j.jbc.2022.102774 PMC9830226 36481270

[168] Li Y et al. The mTORC2/AKT/VCP axis is associated with quality control of the stalled translation of poly (GR) dipeptide repeats in C9-ALS/FTD. J Biol Chem, 102995 (2023). 10.1016/j.jbc.2023.102995 PMC10011831 36764521

[169] Kwon I (2014). Poly-dipeptides encoded by the C9orf72 repeats bind nucleoli, impede RNA biogenesis, and kill cells. Science.

[170] Wen X (2014). Antisense proline-arginine RAN dipeptides linked to C9ORF72-ALS/FTD form toxic nuclear aggregates that initiate in vitro and in vivo neuronal death. Neuron.

[171] Lee KH (2016). C9orf72 Dipeptide repeats impair the Assembly, Dynamics, and function of Membrane-Less Organelles. Cell.

[172] Kanekura K (2016). Poly-dipeptides encoded by the C9ORF72 repeats block global protein translation. Hum Mol Genet.

[173] Hartmann H et al. Proteomics and C9orf72 neuropathology identify ribosomes as poly-GR/PR interactors driving toxicity. Life Sci Alliance 1 (2018). 10.26508/lsa.201800070 PMC6238541 30456350

[174] Moens TG (2019). C9orf72 arginine-rich dipeptide proteins interact with ribosomal proteins in vivo to induce a toxic translational arrest that is rescued by eIF1A. Acta Neuropathol.

[175] Zhang Y-J (2018). Poly (GR) impairs protein translation and stress granule dynamics in C9orf72-associated frontotemporal dementia and amyotrophic lateral sclerosis. Nat Med.

[176] Loveland AB et al. Ribosome inhibition by C9ORF72-ALS/FTD-associated poly-PR and poly-GR proteins revealed by cryo-EM. Nat Commun 13 (2022). 10.1038/s41467-022-30418-0 PMC9120013 35589706

[177] Lagier-Tourenne C, Polymenidou M, Cleveland DW (2010). TDP-43 and FUS/TLS: emerging roles in RNA processing and neurodegeneration. Hum Mol Genet.

[178] Kwiatkowski T (2009). Mutations in the FUS/TLS gene on chromosome 16 cause familial amyotrophic lateral sclerosis. Science.

[179] Shang Y, Huang EJ (2016). Mechanisms of FUS mutations in familial amyotrophic lateral sclerosis. Brain Res.

[180] Deng H, Gao K, Jankovic J (2014). The role of FUS gene variants in neurodegenerative diseases. Nat Reviews Neurol.

[181] Deng HX (2010). FUS-immunoreactive inclusions are a common feature in sporadic and non‐SOD1 familial amyotrophic lateral sclerosis. Ann Neurol.

[182] Lagier-Tourenne C (2012). Divergent roles of ALS-linked proteins FUS/TLS and TDP-43 intersect in processing long pre-mRNAs. Nat Neurosci.

[183] Hoell JI (2011). RNA targets of wild-type and mutant FET family proteins. Nat Struct Mol Biol.

[184] Takahama K (2013). Regulation of telomere length by G-quadruplex telomere DNA-and TERRA-binding protein TLS/FUS. Chem Biol.

[185] Iko Y (2004). Domain architectures and characterization of an RNA-binding protein, TLS. J Biol Chem.

[186] Takeda J-i, Masuda A, Ohno K (2017). Six GU-rich (6GUR) FUS-binding motifs detected by normalization of CLIP-seq by nascent-seq. Gene.

[187] Patel A (2015). A liquid-to-solid phase transition of the ALS protein FUS accelerated by disease mutation. Cell.

[188] Portz B, Lee BL, Shorter J (2021). FUS and TDP-43 phases in health and disease. Trends Biochem Sci.

[189] Kato M (2012). Cell-free formation of RNA granules: low complexity sequence domains form dynamic fibers within hydrogels. Cell.

[190] Birsa N (2021). FUS-ALS mutants alter FMRP phase separation equilibrium and impair protein translation. Sci Adv.

[191] Ohashi R, Shiina N (2020). Cataloguing and selection of mRNAs localized to dendrites in neurons and regulated by RNA-binding proteins in RNA granules. Biomolecules.

[192] Kamelgarn M et al. ALS mutations of FUS suppress protein translation and disrupt the regulation of nonsense-mediated decay. *Proceedings of the National Academy of Sciences* 115, E11904-E11913 (2018). 10.1073/pnas.1810413115 PMC6304956 30455313

[193] Yasuda K (2013). The RNA-binding protein fus directs translation of localized mRNAs in APC-RNP granules. J Cell Biol.

[194] Yasuda K, Clatterbuck-Soper SF, Jackrel ME, Shorter J, Mili S (2017). FUS inclusions disrupt RNA localization by sequestering kinesin-1 and inhibiting microtubule detyrosination. J Cell Biol.

[195] López-Erauskin J (2018). ALS/FTD-linked mutation in FUS suppresses intra-axonal protein synthesis and drives disease without nuclear loss-of-function of FUS. Neuron.

[196] François-Moutal L et al. Structural insights into TDP-43 and effects of post-translational modifications. Front Mol Neurosci, 301 (2019). 10.3389/fnmol.2019.00301 PMC6934062 31920533

[197] Ratti A, Buratti E (2016). Physiological functions and pathobiology of TDP-43 and FUS/TLS proteins. J Neurochem.

[198] Arai T (2006). TDP-43 is a component of ubiquitin-positive tau-negative inclusions in frontotemporal lobar degeneration and amyotrophic lateral sclerosis. Biochem Bioph Res Co.

[199] Neumann M (2006). Ubiquitinated TDP-43 in frontotemporal lobar degeneration and amyotrophic lateral sclerosis. Science.

[200] Buratti E (2015). Functional significance of TDP-43 mutations in disease. Adv Genet.

[201] Suk TR, Rousseaux MW (2020). The role of TDP-43 mislocalization in amyotrophic lateral sclerosis. Mol neurodegeneration.

[202] Lukavsky PJ (2013). Molecular basis of UG-rich RNA recognition by the human splicing factor TDP-43. Nat Struct Mol Biol.

[203] Buratti E (2005). TDP-43 binds heterogeneous nuclear ribonucleoprotein A/B through its C-terminal tail: an important region for the inhibition of cystic fibrosis transmembrane conductance regulator exon 9 splicing. J Biol Chem.

[204] Jain S (2016). ATPase-modulated stress granules contain a diverse proteome and substructure. Cell.

[205] Udan-Johns M (2014). Prion-like nuclear aggregation of TDP-43 during heat shock is regulated by HSP40/70 chaperones. Hum Mol Genet.

[206] Ling S-C, Polymenidou M, Cleveland DW (2013). Converging mechanisms in ALS and FTD: disrupted RNA and protein homeostasis. Neuron.

[207] Johnson BS (2009). TDP-43 is intrinsically aggregation-prone, and amyotrophic lateral sclerosis-linked mutations accelerate aggregation and increase toxicity. J Biol Chem.

[208] Majumder P (2012). TDP-43 regulates the mammalian spinogenesis through translational repression of Rac1. Acta Neuropathol.

[209] Majumder P, Chu J-F, Chatterjee B, Swamy K, Shen C-K (2016). J. Co-regulation of mRNA translation by TDP-43 and Fragile X syndrome protein FMRP. Acta Neuropathol.

[210] Coyne AN (2014). Futsch/MAP1B mRNA is a translational target of TDP-43 and is neuroprotective in a Drosophila model of amyotrophic lateral sclerosis. J Neurosci.

[211] Coyne AN (2017). Post-transcriptional inhibition of Hsc70-4/HSPA8 expression leads to synaptic vesicle cycling defects in multiple models of ALS. Cell Rep.

[212] Neelagandan N (2019). TDP-43 enhances translation of specific mRNAs linked to neurodegenerative disease. Nucleic Acids Res.

[213] Russo A (2017). Increased cytoplasmic TDP-43 reduces global protein synthesis by interacting with RACK1 on polyribosomes. Hum Mol Genet.

[214] Charif SE, Luchelli L, Vila A, Blaustein M, Igaz LM (2020). Cytoplasmic expression of the ALS/FTD-related protein TDP-43 decreases global translation both in vitro and in vivo. Front Cell Neurosci.

[215] Lehmkuhl EM (2021). TDP-43 proteinopathy alters the ribosome association of multiple mRNAs including the glypican Dally-like protein (dlp)/GPC6. Acta Neuropathol Commun.

[216] Freibaum BD, Chitta RK, High AA, Taylor JP (2010). Global analysis of TDP-43 interacting proteins reveals strong association with RNA splicing and translation machinery. J Proteome Res.

[217] Higashi S (2013). TDP-43 associates with stalled ribosomes and contributes to cell survival during cellular stress. J Neurochem.

[218] Gao J (2021). Translational regulation in the brain by TDP-43 phase separation. J Cell Biol.

[219] Altman T et al. Axonal TDP-43 condensates drive neuromuscular junction disruption through inhibition of local synthesis of nuclear encoded mitochondrial proteins. Nat Commun 12 (2021). 10.1038/s41467-021-27221-8 PMC8617040 34824257

[220] Tank EM (2018). Abnormal RNA stability in amyotrophic lateral sclerosis. Nat Commun.

[221] Clarke JP, Thibault PA, Salapa HE, Levin MC (2021). A comprehensive analysis of the role of hnRNP A1 function and dysfunction in the pathogenesis of neurodegenerative disease. Front Mol Biosci.

[222] Liu Y, Shi SL (2021). The roles of hnRNP A2/B1 in RNA biology and disease. Wiley Interdisciplinary Reviews: RNA.

[223] Siomi H, Dreyfuss GA (1995). Nuclear-localization domain in the Hnrnp A1 protein. J Cell Biol.

[224] Molliex A (2015). Phase separation by low complexity domains promotes stress granule assembly and drives pathological fibrillization. Cell.

[225] Ryan VH (2018). Mechanistic view of hnRNPA2 low-complexity domain structure, interactions, and phase separation altered by mutation and arginine methylation. Mol Cell.

[226] Guil S, Long JC, Cáceres JF (2006). hnRNP A1 relocalization to the stress granules reflects a role in the stress response. Mol Cell Biol.

[227] Kim HJ (2013). Mutations in prion-like domains in hnRNPA2B1 and hnRNPA1 cause multisystem proteinopathy and ALS. Nature.

[228] Lu J (2020). CryoEM structure of the low-complexity domain of hnRNPA2 and its conversion to pathogenic amyloid. Nat Commun.

[229] Bonnal S (2005). Heterogeneous nuclear ribonucleoprotein A1 is a novel internal ribosome entry site trans-acting factor that modulates alternative initiation of translation of the fibroblast growth factor 2 mRNA. J Biol Chem.

[230] Jung Y (2020). BDNF-induced local translation of GluA1 is regulated by HNRNP A2/B1. Sci Adv.

[231] Sheng J, Xu Z (2016). Three decades of research on angiogenin: a review and perspective. Acta Biochim Biophys Sin.

[232] Russo N, Nobile V, Di Donato A, Riordan JF, Vallee BL. The C-terminal region of human angiogenin has a dual role in enzymatic activity. *Proceedings of the National Academy of Sciences* 93, 3243–3247 (1996). 10.1073/pnas.93.8.3243 PMC39590 8622921

[233] Leland PA, Staniszewski KE, Park C, Kelemen BR, Raines RT (2002). The ribonucleolytic activity of angiogenin. Biochemistry-Us.

[234] Moroianu J, Riordan JF. Nuclear translocation of angiogenin in proliferating endothelial cells is essential to its angiogenic activity. *Proceedings of the National Academy of Sciences* 91, 1677–1681 (1994). 10.1073/pnas.91.5.1677 PMC43226 8127865

[235] Moroianu J, Riordan JF (1994). Identification of the nucleolar targeting signal of human angiogenin. Biochem Bioph Res Co.

[236] Sarangdhar MA, Allam R (2021). Angiogenin (ANG)—ribonuclease inhibitor (RNH1) system in protein synthesis and disease. Int J Mol Sci.

[237] Gagliardi S (2019). A novel nonsense angiogenin mutation is associated with Alzheimer disease. Alzheimer Disease & Associated Disorders.

[238] Prehn JH, Jirström E (2020). Angiogenin and tRNA fragments in Parkinson’s disease and neurodegeneration. Acta Pharmacol Sin.

[239] Sheng J, Yu W, Gao X, Xu Z, Hu GF (2014). Angiogenin stimulates ribosomal RNA transcription by epigenetic activation of the ribosomal DNA promoter. J Cell Physiol.

[240] Hoang TT, Raines RT (2017). Molecular basis for the autonomous promotion of cell proliferation by angiogenin. Nucleic Acids Res.

[241] Monti DM (2009). Characterization of the angiogenic activity of zebrafish ribonucleases. FEBS J.

[242] Lyons SM, Fay MM, Akiyama Y, Anderson PJ, Ivanov P (2017). RNA biology of angiogenin: current state and perspectives. Rna Biol.

[243] Czech A, Wende S, Mörl M, Pan T, Ignatova Z (2013). Reversible and rapid transfer-RNA deactivation as a mechanism of translational repression in stress. PLoS Genet.

[244] Yamasaki S, Ivanov P, Hu G-f, Anderson P (2009). Angiogenin cleaves tRNA and promotes stress-induced translational repression. J Cell Biol.

[245] Fu H (2009). Stress induces tRNA cleavage by angiogenin in mammalian cells. Febs Lett.

[246] Hogg MC (2020). 5′ ValCAC tRNA fragment generated as part of a protective angiogenin response provides prognostic value in amyotrophic lateral sclerosis. Brain Commun.

[247] Ivanov P, Emara MM, Villen J, Gygi SP, Anderson P (2011). Angiogenin-induced tRNA fragments inhibit translation initiation. Mol Cell.

[248] Emara MM (2010). Angiogenin-induced tRNA-derived stress-induced RNAs promote stress-induced stress granule assembly. J Biol Chem.

[249] Subramanian V, Crabtree B, Acharya KR (2008). Human angiogenin is a neuroprotective factor and amyotrophic lateral sclerosis associated angiogenin variants affect neurite extension/pathfinding and survival of motor neurons. Hum Mol Genet.

[250] Fernández-Gómez A, Izquierdo JM (2022). The multifunctional faces of T-Cell Intracellular Antigen 1 in Health and Disease. Int J Mol Sci.

[251] Rayman JB, Kandel ER (2017). TIA-1 is a functional prion-like protein. Cold Spring Harb Perspect Biol.

[252] Gilks N (2004). Stress granule assembly is mediated by prion-like aggregation of TIA-1. Mol Biol Cell.

[253] Mackenzie IR et al. TIA1 mutations in amyotrophic lateral sclerosis and frontotemporal dementia promote phase separation and alter stress granule dynamics. *Neuron* 95, 808–816. e809 (2017). 10.1016/j.neuron.2017.07.025 PMC5576574 28817800

[254] Sekiyama N et al. ALS mutations in the TIA-1 prion-like domain trigger highly condensed pathogenic structures. *Proceedings of the National Academy of Sciences* 119, e2122523119 (2022). 10.1073/pnas.2122523119 PMC9499527 36112647

[255] Piecyk M (2000). TIA-1 is a translational silencer that selectively regulates the expression of TNF-α. EMBO J.

[256] Dixon DA (2003). Regulation of cyclooxygenase-2 expression by the translational silencer TIA-1. J Exp Med.

[257] Carrascoso I, Sánchez-Jiménez C, Izquierdo JM (2014). Long-term reduction of T-cell intracellular antigens leads to increased beta-actin expression. Mol Cancer.

[258] Kawai T (2006). Translational control of cytochrome c by RNA-binding proteins TIA-1 and HuR. Mol Cell Biol.

[259] Rodrigues DC (2016). MECP2 is post-transcriptionally regulated during human neurodevelopment by combinatorial action of RNA-binding proteins and miRNAs. Cell Rep.

[260] de López I (2005). Identification and functional outcome of mRNAs associated with RNA-binding protein TIA-1. Mol Cell Biol.

[261] Carrascoso I, Sánchez-Jiménez C, Izquierdo JM (2014). Genome-wide profiling reveals a role for T-cell intracellular antigens TIA1 and TIAR in the control of translational specificity in HeLa cells. Biochem J.

[262] Byres LP (2021). Identification of TIA1 mRNA targets during human neuronal development. Mol Biol Rep.

[263] Filbeck S, Cerullo F, Pfeffer S, Joazeiro CA (2022). Ribosome-associated quality-control mechanisms from bacteria to humans. Mol Cell.

[264] Shao S, Brown A, Santhanam B, Hegde RS (2015). Structure and assembly pathway of the ribosome quality control complex. Mol Cell.

[265] Chu J et al. A mouse forward genetics screen identifies LISTERIN as an E3 ubiquitin ligase involved in neurodegeneration. *Proceedings of the National Academy of Sciences* 106, 2097–2103 (2009). 10.1073/pnas.0812819106 PMC2650114 19196968

[266] Martin PB (2020). NEMF mutations that impair ribosome-associated quality control are associated with neuromuscular disease. Nat Commun.

[267] Ahmed A (2021). Biallelic loss-of-function variants in NEMF cause central nervous system impairment and axonal polyneuropathy. Hum Genet.

[268] Sonobe Y et al. Translation of dipeptide repeat proteins in C9ORF72-ALS/FTD through unique and redundant AUG initiation codons. *bioRxiv* (2022). 10.7554/eLife.83189 PMC10541178 37675986

[269] Burguete AS (2015). GGGGCC microsatellite RNA is neuritically localized, induces branching defects, and perturbs transport granule function. Elife.

[270] Genuth NR, Barna M (2018). Heterogeneity and specialized functions of translation machinery: from genes to organisms. Nat Rev Genet.

[271] Sun S et al. Translational profiling identifies a cascade of damage initiated in motor neurons and spreading to glia in mutant SOD1-mediated ALS. *Proceedings of the National Academy of Sciences* 112, E6993–E7002 (2015). 10.1073/pnas.1520639112 PMC4687558 26621731

[272] Zeng H et al. Spatially resolved single-cell translatomics at molecular resolution. Science. 2023;380(6652):eadd3067. 10.1126/science.add3067 PMC11146668 37384709

